# Endothelial Insulin Resistance of Freshly Isolated Arterial Endothelial Cells From Radial Sheaths in Patients With Suspected Coronary Artery Disease

**DOI:** 10.1161/JAHA.118.010816

**Published:** 2019-03-19

**Authors:** Nobuyuki Masaki, Yasuo Ido, Toshiyuki Yamada, Youhei Yamashita, Takumi Toya, Bonpei Takase, Naomi M. Hamburg, Takeshi Adachi

**Affiliations:** ^1^ Department of Intensive Care Medicine National Defense Medical College Tokorozawa Japan; ^2^ Department of Cardiology National Defense Medical College Tokorozawa Japan; ^3^ Department of Cardiovascular Surgery Keio University Graduate School of Medicine Tokyo Japan; ^4^ The Whitaker Cardiovascular Institute Department of Medicine Boston University School of Medicine Boston MA

**Keywords:** arterial stiffness, endothelial nitric oxide synthase, insulin resistance, Clinical Studies, Endothelium/Vascular Type/Nitric Oxide, Vascular Biology, Oxidant Stress

## Abstract

**Background:**

Endothelial insulin resistance is insulin‐insensitivity in the vascular endothelium and can be observed in experimental models. This study aimed to investigate endothelial insulin resistance in patients with suspected coronary artery disease. To this end, a novel method of obtaining freshly isolated arterial endothelial cells from a radial catheter sheath was developed.

**Methods and Results:**

Freshly isolated arterial endothelial cells were retrieved from catheter sheaths placed in radial arteries for coronary angiography (n=69, patient age 64±12 years). The endothelial cells were divided into groups for incubation with or without insulin, vascular endothelial growth factor, or acetylcholine. The intensity of phosphorylated endothelial nitric oxide synthase at Ser1177 (p‐eNOS) was quantified by immunofluorescence microscopy. The percentage increase of insulin‐induced phosphorylated endothelial nitric oxide synthase correlated negatively with derivatives of reactive oxygen metabolites, an oxidative stress test (*r*=−0.348, n=53, *P*=0.011), E/E′, an index of left ventricular diastolic dysfunction in Doppler echocardiography (ρ=−0.374, n=49, *P*=0.008), and log‐transformed brain natriuretic peptide (*r*=−0.266, n=62, *P*=0.037). Furthermore, percentage increase of insulin‐induced p‐eNOS was an independent factor for the cardio‐ankle vascular index (standardized coefficient β=−0.293, n=42, *P*=0.021) in the multivariate regression analysis of adaptive least absolute shrinkage and selection operator.

**Conclusions:**

Our results suggested that endothelial insulin resistance is associated with oxidative stress, left ventricular diastolic dysfunction, heart failure, and arterial stiffness.

**Clinical Perceptive**

**What Is New?**

- We developed a new non‐invasive method to collect human freshly isolated arterial endothelial cells from radial catheter sheaths, which are disposable devices for coronary angiography.
- Using the freshly isolated arterial endothelial cells, endothelial insulin resistance, defined as reduced insulin‐induced endothelial nitric oxide synthase activation via insulin receptor substrate‐1/phosphatidylinositol 3‐kinase/protein kinase B/endothelial nitric oxide synthase signaling. was assessed by immunofluorescent microscopy.

**What Are the Clinical Implications?**

- Endothelial insulin resistance was associated with non‐diabetic factors including oxidative stress, heart failure, and arterial stiffness assessed by cardio‐ankle vascular index.

## Introduction

Insulin resistance (IR) is classically defined as the impaired action of insulin in organs responsible for glucose metabolism, including adipose tissue, skeletal muscle, and the liver.^1^ In IR, insulin‐mediated glucose uptake is reduced in muscles and adipocytes. Moreover, hepatic gluconeogenesis and lipogenesis are increased, whereas lipolysis occurs in adipose tissue. However, a more comprehensive concept includes insulin‐insensitivity in other organs that are regulated by insulin but not directly involved in the control of blood glucose levels.^2^

Endothelial IR is insulin‐insensitivity in the vascular endothelium and is considered peripheral IR.^3^ Insulin directly stimulates the release of nitric oxide (NO) from the endothelium by activating insulin receptor substrate (IRS)‐1, which leads to phosphatidylinositol 3‐kinase (PI3K)‐protein kinase B (Akt)‐mediated phosphorylation of endothelial NO synthase (eNOS).^4^, ^5^, ^6^, ^7^ NO promotes vasodilatation and prevents leukocyte adhesion,^8^ thrombocyte aggregation,^9^ and smooth muscle cell proliferation.^10^ Therefore, when the action of insulin is reduced in the endothelium, it is thought to be associated with endothelial dysfunction and other vascular diseases.

At present, the definition of endothelial IR is almost equal to deterioration of IRS‐1/PI3K/Akt/eNOS signaling, which can be observed only in experimental models using endothelial cells or animals.^2^ The concept of endothelial IR has not been clinically established because only a small number of studies have shown the actual response to insulin in endothelial cells of patients with metabolic disorders to date.^11^, ^12^ Many clinical studies have reported that IR decreases NO synthesis in the endothelium. However, the assessments in these studies were based on metabolic IR, for example the homeostatic model assessment of insulin resistance.^13^ Thus, if possible, it is preferable to evaluate endothelial IR for this purpose. Therefore, we have developed a novel method of obtaining endothelial cells from patients. We collected arterial endothelial cells from radial catheter sheaths, which are disposable devices for coronary angiography. This does not require any additional invasive procedures and can be used in many patients.

The aim of this study was to explore the influential factors of endothelial IR using our novel method because IRS‐1/PI3K/Akt/eNOS pathway can be deteriorated by various factors. Our hypothesis is that endothelial IR may occur in patients with not only diabetes mellitus but also cardiovascular disease which can cause endothelial dysfunction such as heart failure and atherosclerosis. We considered eNOS responses to insulin as an index of endothelial IR,^11^, ^12^ and compared them with laboratory examination, echocardiographic parameters, and the cardio‐ankle vascular index (CAVI). Lower amplification of insulin‐induced phosphorylated endothelial NO synthase at Ser1177 (p‐eNOS) has been shown to be an indicator of insufficient NO production.^12^ CAVI is an indicator of arterial stiffness that excludes the effect of blood pressure at the time of the measurement.^14^ In addition, we assessed eNOS responses to vascular endothelial growth factor (VEGF) and acetylcholine.

## Materials and Methods

The authors declare that all supporting data are available within the article and its online supplementary files. Additional methods and results can be found in Data S1.

### Subjects

Patients who underwent elective cardiac catheterization from January 2017 to December 2017 at the National Defense Medical College Tokorozawa, Japan were enrolled in the study. Exclusion criteria included ongoing treatment for malignant tumor and hemodialysis. The study protocol was approved by the National Defense Medical College Review Board, and all participants provided written informed consent.

Hypertension was identified as blood pressure above 140/90 mm Hg or as receiving medication for the condition. Diabetes mellitus (DM) was diagnosed as fasting blood glucose >126 mg/dL or the use of insulin or oral hypoglycemic agents. Hyperlipidemia was defined as total cholesterol >220 mg/dL, low‐density lipoprotein cholesterol >140 mg/dL, or receiving anti‐hyperlipidemic medication. Estimated glomerular filtration rate (eGFR) was calculated using the Modification of Diet in Renal Disease equation modified for a Japanese population.^15^

### Laboratory Examination

Blood samples were drawn through a guiding sheath during coronary angiography without the administration of heparin or nitroglycerin, collected into plain tubes, and refrigerated immediately. Serum was obtained by centrifugation at 1610*g* for 10 minutes at 4°C. Derivatives of reactive oxygen metabolites (d‐ROMs) were measured in serum using the reactive oxygen metabolites free radical test (Dacron International, Grosseto, Italy). The d‐ROMs test was used to quantify total hydroperoxide levels by measuring the ability of transition metals to catalyze the formation of free radicals. Oxidized N,N‐diethyl‐para‐phenylenediamine was detected spectrophotometrically at 505 nm.^16^, ^17^ One unit of d‐ROMs (U‐CARR) corresponds to the amount of hydroperoxide that can be converted by superoxide dismutase to approximately 0.08 mg/dL H_2_O_2_. Homeostatic model assessment of insulin resistance was calculated from fasting insulin levels, as previously described.^18^

### Coronary Angiography

Coronary angiography was performed with a 4 Fr catheter system. Angiograms were taken from at least 4 standard projections for each right and left coronary artery. Coronary artery disease (CAD) was defined as the presence of coronary stenosis of >75% in at least 1 coronary vessel in the angiogram, or a past history of myocardial infarction, percutaneous coronary intervention, or coronary artery bypass grafting surgery.

### Physiological Tests

Cardio‐ankle vascular index (CAVI) was obtained using a VaSera CAVI instrument (Fukuda Denshi Co, Ltd, Tokyo), equipped with electrocardiography, phonocardiography, and mechanocardiography functions. CAVI was recorded in patients after 5 minutes of rest in the supine position. The calculation of CAVI is based on blood pressure and heart‐ankle pulse wave velocity, monitoring of heart sounds, and electrocardiography. Heart‐ankle pulse wave velocity was calculated by dividing the distance from the aortic valve to the ankle artery by the sum of the time intervals between aortic valve closure sound (first part of the second heart sound) and the notch of the brachial pulse wave, and between the rise of the brachial pulse wave and the ankle pulse wave. CAVI was determined using the following formula,

CAVI=a[2ρ/(Ps−Pd)×ln(Ps/Pd)×ha PWV2]+b

where Ps and Pd are systolic and diastolic blood pressure, respectively; ρ is blood density; and a and b are constants. CAVI was taken as the average of the right and left CAVI values.

**Figure 1:**
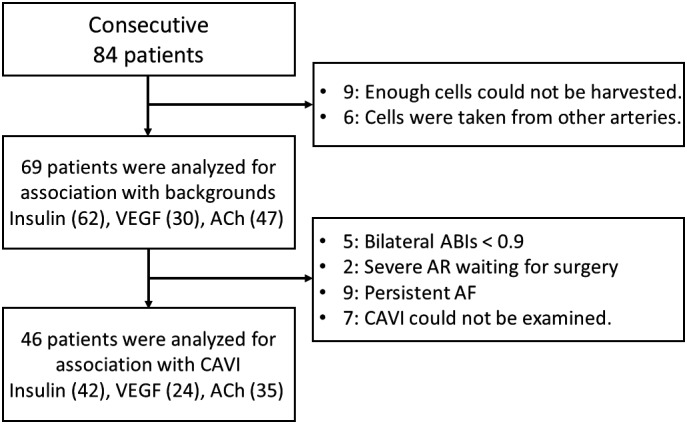
Enrollment of the patients. Exclusion criteria are described. Of 84 consecutive patients undergoing coronary angiography, data from 69 patients were analyzed for comparison between the cell study and traditional cardiovascular risk factors. The numbers in parentheses represent patients whose endothelial cells could be examined under biochemical stimulation. Among them, 46 patients met the inclusion criteria for CAVI. ABI indicates ankle brachial index; AF, atrial fibrillation; AR, aortic valve regurgitation; CAVI, cardio‐ankle vascular index; VEGF, vascular endothelial growth factor.

A CAVI score of <8.0 is supposed to be normal, whereas a value <9.0 but ≥8.0 is considered “borderline”. A CAVI ≥9.0 leads to a diagnosis of suspected arteriosclerosis.^14^, ^19^ For the CAVI evaluation, we excluded patients with severe aortic insufficiency, bilateral ankle‐brachial index <0.9, or persistent atrial fibrillation (Figure 1) because it is difficult to obtain accurate measurements in such patients.^20^

### Echocardiography

Transthoracic echocardiography was performed by trained sonographers. Peak early diastolic transmitral flow velocity (E) and peak late diastolic transmitral flow velocity (A) phases of mitral inflow were recorded at the mitral valve leaflet tips from an apical 4‐chamber view using pulsed Doppler echocardiography. Deceleration time was derived from the early filling wave and left ventricular (LV) myocardial velocity was evaluated at the septal mitral valve annulus. The peak early diastolic annular velocity (E′) was measured using tissue‐Doppler imaging. The ratio of early mitral filling velocity (E) to E′ (E/E′) was calculated as an index of LV diastolic dysfunction. The ejection fraction was measured using echocardiography with the Teichholz method or biplane modified Simpson method if appropriate.

### Peripheral Endothelial Cell Collection

An inner dilator of a radial catheter sheath used for coronary angiography was extracted under a sterile technique (Medikit Co, Ltd, Japan) as follows. A right wrist was sterilized. A radial artery was punctured by a thin needle with inner lumen. Then, a guidewire was placed into the artery through the needle. A 4 Fr radial catheter sheath was inserted into radial artery along with the guidewire. The inner dilator of the sheath was extracted together with the guidewire from the radial artery. In the laboratory, endothelial cells adherent to the disposable device were removed by washing with an erythrocyte lysing kit (R&D Systems) and the pellet was obtained by centrifugation. The pellet was washed again with endothelial basal medium (EBM‐2; Lonza, Basel, Switzerland) and plated on poly‐L‐lysine‐coated microscope slides (Sigma, St. Louis, MO). After the incubation in 5% CO_2_ 37° for 30 minutes, the endothelial cells were treated with or without insulin (WAKO: 100 nmol/L, 30 minutes), recombinant human vascular endothelial growth factor (WAKO: 20 ng/mL, 30 minutes), or acetylcholine (Sigma: 1 μmol/L, 15 minutes). Next, the cells were fixed immediately in 4% paraformaldehyde and the slides were washed in PBS, dried and stored at −80°C until further processing. The process from harvesting cells to fixation takes 90 minutes.

### Assessment of Protein Expression by Quantitative Immunofluorescence

Fixed sample slides were thawed and rehydrated with PBS containing 50 mmol/L glycine (Sigma) for 10 minutes. The cells on the slides were permeabilized with 0.1% Triton X‐100, and non‐specific binding sites were blocked with 0.5% BSA. The slides were incubated overnight at 4°C with primary antibodies against the following targets: phosphorylated eNOS at ser1177 (p‐eNOS) (1:200 dilution; GeneTex, Inc, Irvine, CA), total eNOS (1:100 dilution; Millipore, MA), vascular cell adhesion molecule‐1 (1:200 dilution; Abcam, Cambridge, MA), vascular endothelial cadherin (1:50; Santa‐Cruz, Dallas, TX). All slides were double‐stained with anti–von Willebrand Factor (vWF) antibody (1:300 dilution; Thermo Fisher Scientific) for identification of endothelial cells. After incubation, the slides were washed and incubated for 3 hours at 37°C with corresponding Alexa Fluor‐488 and Alexa Fluor‐594 antibodies (1:200 dilution; Invitrogen, Carlsbad, CA). The slides were washed 3 times and mounted under glass coverslips with Vectashield Antifade Mounting Medium containing DAPI for nuclear identification (Vector Laboratories, Burlingame, CA). For each batch of patient‐derived cells, we stained a control slide of human aortic endothelial cells at each staining.

The immunofluorescence intensity was quantified by modifying methods reported previously.^11^, ^12^ Slide images were obtained with a fluorescence microscope (BZ‐X700, KEYENCE, Japan) at ×40 magnification. The exposure time was constant at 200 ms for p‐eNOS. Fluorescence intensity was quantified by a software program (KEYENCE Corp., Osaka, Japan). Image intensity was corrected for background fluorescence by subtraction. For each protein of interest, fluorescence intensity was quantified in 20 cells from each patient and the means were obtained. Intensity is expressed in arbitrary units (au), which is the percentage of the average fluorescence intensity from the patient sample to the average fluorescence intensity of the human aortic endothelial cells slide stained at the same time. This formula is used to adjust deviations under staining conditions. Quantification was performed with blinding to the identity of the subject.

The percentage change of p‐eNOS by each stimulus (insulin, VEGF, acetylcholine) was calculated as follows:

$$\begin{array}{cc} & \text{Percent change of p‐eNOS}\;(\%)=100\times (\text{stimulated p‐eNOS}\\ & -\text{basal p‐eNOS})/\text{basal p‐eNOS} \end{array}$$

where basal p‐eNOS is the intensity of p‐eNOS without stimulation.

### Statistical Analyses

The distribution of continuous clinical characteristics and measurements were evaluated by examining a histogram and applying the Shapiro–Wilk test. The 2‐group comparisons were performed with *t* test or Mann–Whitney *U* test as appropriate. Categorical clinical characteristics were compared using χ^2^ testing or Fisher exact test if appropriate. The correlation coefficient of 2 variables of normal distribution was obtained with Pearson method. Spearman method was used if at least 1 variable of non‐normal distribution was included. The paired *t* test was used for paired samples for immunofluorescent intensities before and after serum stimulation.

Univariate and multivariate regression analyses were performed to identify independent variables associated with CAVI scores from clinical features and the results of cell experiments. In the multivariate analysis, traditional cardiovascular risk factors and the independent factors correlating with CAVI (<0.1) in the univariate analysis were included in a crude model (model 1). Next, backward stepwise method was used to select effective explanatory variables from the variables used in model 1 (model 2). In addition, we performed the adaptive least absolute shrinkage and selection operator (Lasso) regression analysis, which is currently considered to obtain a better‐fitting model for small size samples (model 3).^21^ d‐ROMs were not included in the regression models because there was an insufficient number of patients. Statistical analyses were performed using SPSS version 22.0 (SPSS Japan, Tokyo), and JMP pro. version 13.1.0 (SAS Institute Japan, Tokyo) for the adaptive Lasso regression analysis.

Summary data are presented as means±SDs for variables of normal distribution or median (1st quartile, 3rd quartile) for those of non‐normal distribution. In all analyses, *P*<0.05 was considered statistically significant.

## Results

### Subjects

A total of 84 consecutive patients undergoing coronary angiography were enrolled in this study (Figure 1). Among them, the data of 69 patients were available for the analysis (Table 1). The study population included 39 patients (57%) with coronary artery disease (CAD). Revascularization had been performed in 25 patients. One patient had previously undergone both coronary artery bypass grafting (CABG) and percutaneous coronary intervention (PCI). In addition, CAVI scores were recorded in 46 patients. The average CAVI score was 8.78±1.71 (Table 2).

**Table 1:** Clinical Characteristics

	Patients (n=69)
Age, y	64±12
Sex (women/men), n (%)	19/50 (28/72)
BMI, kg/m^2^	23±3
Hypertension, n (%)	47 (68)
Hyperlipidemia, n (%)	43 (62)
Diabetes mellitus, n (%)	22 (32)
Current smoking, n (%)	25 (36)
Previous conditions	
CAD, n (%)	39 (57)
OMI, n (%)	13 (19)
CABG, n (%)	5 (7)
PCI, n (%)	21 (30)
Persistent AF, n (%)	9 (13)
DCM, n (%)	8 (12)
HCM, n (%)	2 (3)
Aortic aneurysm, n (%)	5 (7)
Valvular heart disease, n (%)	9 (13)
Hospital admission for heart failure, n (%)	17 (25)
Medications	
Beta‐blocker, n (%)	36 (52)
ACE inhibitor, n (%)	12 (17)
ARB, n (%)	27 (39)
Calcium channel blocker, n (%)	22 (32)
Furosemide, n (%)	17 (25)
Spironolactone, n (%)	13 (19)
Statins, n (%)	42 (61)
Insulin, n (%)	5 (7)
Warfarin, n (%)	9 (13)
P2Y_12_ inhibitor, n (%)	24 (35)
Aspirin, n (%)	31 (45)

Data are expressed as means or counts (%) as appropriate. ACE indicates angiotensin‐converting enzyme; AF, atrial fibrillation; ARB, angiotensin receptor blocker; BMI, body mass index; CABG, coronary artery bypass surgery; CAD,  coronary artery disease; DCM, dilated cardiomyopathy; HCM, hypertrophic cardiomyopathy; OMI, old myocardial infarction; PCI, percutaneous coronary intervention.

**Table 2:** Experimental Results of p‐eNOS and Laboratory Data

	Patients (n=69)
Endothelial experiments	
Basal p‐eNOS, au	41±22
ΔINS, %*	7±31
ΔVEGF, %†	−0.6±30
ΔACh, %‡	7±39
Physiological examinations	
CAVI§	8.78±1.71
E/E′∥	11 (9, 16)
DCT, ms¶	209±78
EF (%)#	63 (43, 73)
Laboratory data	
WBC, /μL	6091±2041
Hemoglobin, g/dL	14 (13, 15)
Hematocrit, %	40±5
Platelets, ×10^4^/μL	24±7
AST, IU/L	22 (19, 28)
ALT, IU/L	20 (15, 28)
LDL cholesterol, mg/dL	94±31
riglyceride, mg/dL	108 (83, 167)
HDL cholesterol, mg/dL	52±16
Glucose, mg/dL	98 (90, 107)
HbA1C (%)	5.8 (5.6, 6.7)
Uric acid, mg/dL	5.8 (5.0, 7.0)
eGFR, mL/min	68 (54, 79)
CRP, mg/dL	0.3 (0.3, 0.75)
Log_10_BNP	1.73±0.70
d‐ROMs (U.CARR.)#	304±91

Data are expressed as means, medians or counts (%) as appropriate. ALT indicates alanine aminotransferase; AST, aspartate aminotransferase; CAVI, cardio‐ankle vascular index; CRP, C‐reactive protein; DCT, deceleration time; d‐ROMs, derivatives of the reactive oxidative metabolites; E/E′, index of LV diastolic dysfunction; EF, ejection fraction; eGFR, estimated glomerular filtration rate; HbA1C, hemoglobin A1C; HDL, high‐density lipoprotein; LDL, low‐density lipoprotein; Log_10_BNP, log‐transformed brain natriuretic peptide (pg/mL); p‐eNOS, phosphorylated endogenous nitric oxide synthase at Ser1177; WBC, white blood cells; ΔACh, percentage change in acetylcholine‐induced p‐eNOS; ΔINS, percentage change in insulin‐induced p‐eNOS; ΔVEGF, percentage change in VEGF‐induced p‐eNOS.

*n=62.

†n=30.

‡n=47.

§n=46.

∥n=54.

¶n=58.

#n=60.

### Identification of Endothelial Cells

**Figure 2:**
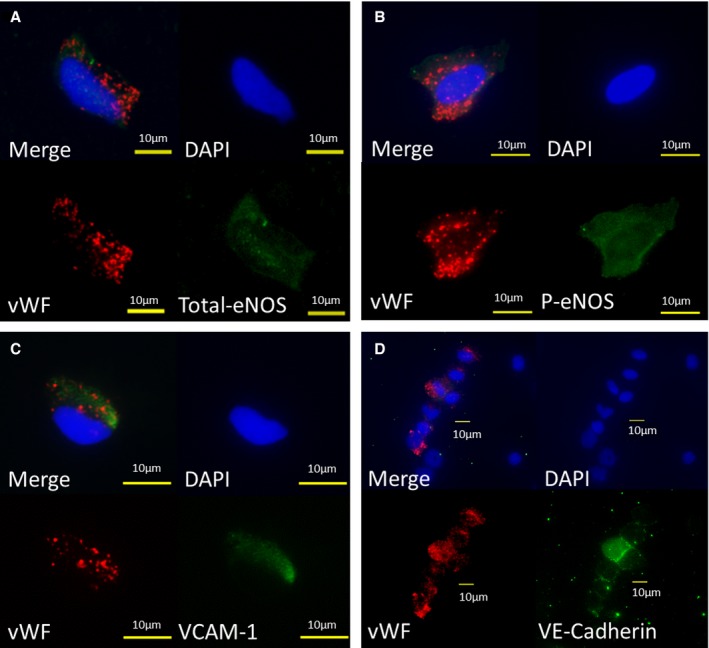
Representative images of the freshly isolated arterial endothelial cell. Red—von Willebrand Factor; Green—total eNOS (**A**), p‐eNOS (**B**), VCAM‐1 (**C**), vascular endothelial‐cadherin (**D**); Blue—DAPI. DAPI indicates 4′,6‐diamidino‐2‐phenylindole stain; eNOS, endogenous nitric oxide synthase; p‐eNOS, phosphorylated‐eNOS at Ser1177; VCAM‐1, vascular cell adhesion molecule‐1; VE‐cadherin, vascular endothelial cadherin; vWF, von Willebrand Factor.

The arterial endothelial cells were identified by microscope by weibel‐palade bodies of vWF staining. The vWF positive cell was also probed by antibodies of total eNOS, and p‐eNOS Ser1177 (Figure 2A and 2B). We found some cells to be positive for anti‐ vascular cell adhesion molecule‐1 antibody (Figure 2C). The image of vascular endothelial‐cadherin was also shown in Figure 2D.

### Quantification of the Immunofluorescent Intensity

The validation of the immunofluorescent quantification was performed using commercialized human umbilical vein endothelial cells. The results are shown in Figures S1 through S3. We compared the results of Western blotting and immunofluorescence using this antibody (Figure S4). There was a positive linear correlation between the intensities evaluated by the 2 modalities.

**Figure 3:**
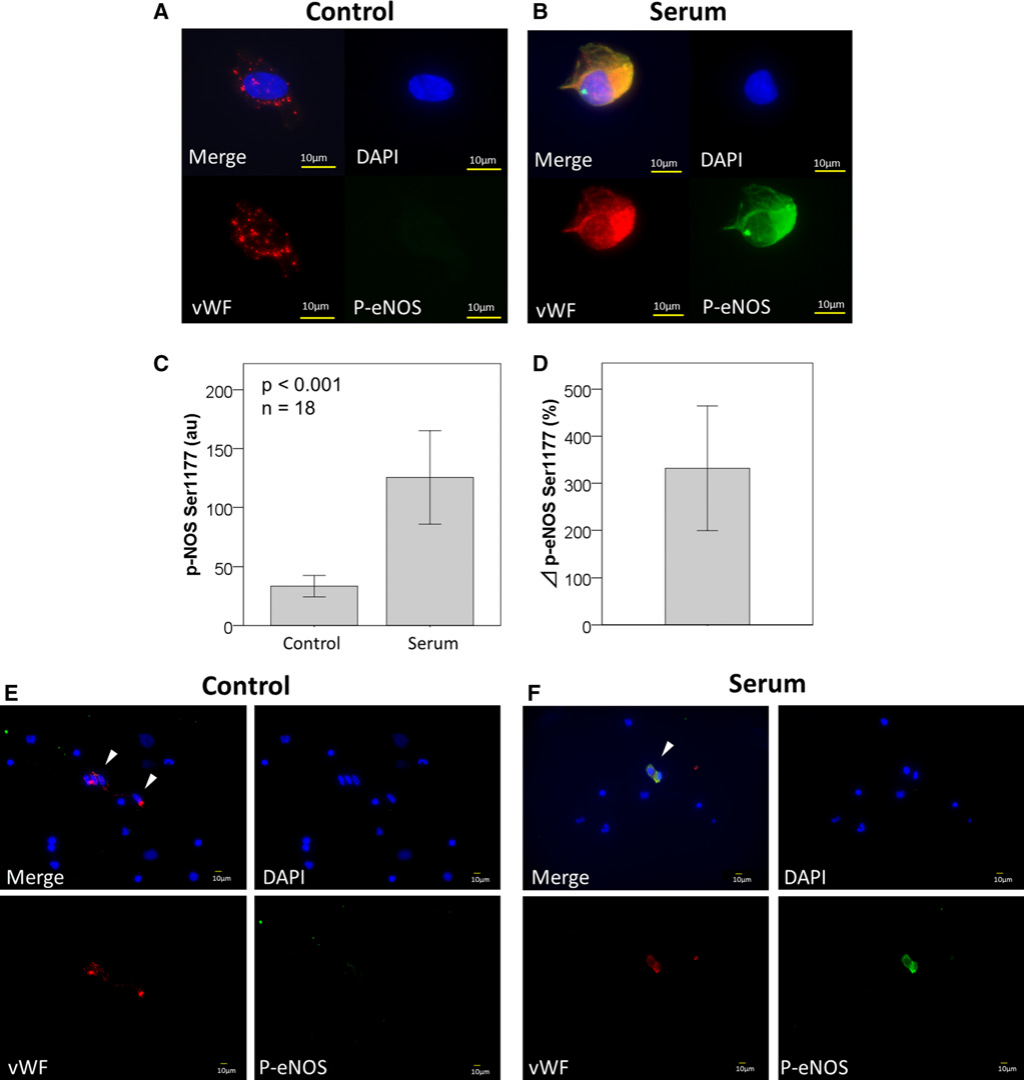
Serum‐stimulation for a positive control. Freshly isolated endothelial cells harvested from a patient was incubated with serum‐free medium as control (**A**) or serum obtained from the patient (**B**) for 30 minutes. The intensity of p‐eNOS (arbritrary unit) was strongly increased by thrombin in the serum (control: 33±20, serum 125±80, n=18; *P*<0.001) (**C**). The % increase of serum‐stimulation was 331±267, which was much higher than insulin, VEGF, acetylcholine (**D**). The images of low magnification (control: **E**; serum‐stimulation: **F**) revealed that the intensity of p‐eNOS was enhanced in only the vWF positive cells. The white arrows indicate the endothelial cells. DAPI indicates 4′,6‐diamidino‐2‐phenylindole stain; p‐eNOS, phosphorylated‐endogenous nitric oxide synthase at Ser1177; VEGF, vascular endothelial growth factor; vWF, von Willebrand Factor.

In addition, we preformed serum‐stimulation to estimate the maximum intensity of p‐eNOS Ser1177. It was previously known that thrombin in serum phosphorylates eNOS.^22^, ^23^, ^24^ In the Western blotting of human umbilical vein endothelial cells, p‐eNOS Ser1177 was increased after augmented p‐Akt Ser473 by human fresh serum. This increase was eliminated by eNOS knockdown (Figures S5 and S6). Therefore, we subsequently cultured freshly isolated endothelial cells harvested from 18 patients with their serum. Figure 3 shows the endothelial cells collected from the same patient (Figure 3A and 3E: control, Figure 3B and 3F: serum‐stimulation). The intensity of p‐eNOS was dramatically increased by serum (Figure 3C and 3D). The p‐eNOS Ser1177 intensity could be a positive control of the experiment.

### Clinical Factors Associated With Basal Phosphorylation of eNOS at Ser1177

Table 3 shows the relationships between the clinical backgrounds of the patients and the immunofluorescence intensity of freshly isolated endothelial cells stained with p‐eNOS antibody. The mean value of basal p‐eNOS measured in arbitrary units (au) was 41±22 (n=69). The quantitated intensity was higher in patients with hyperlipidemia (hyperlipidemia, 46±23, n=43; others, 33±16, n=26; *P*=0.020). In addition, the intensities in endothelial cells correlated positively with log‐transformed brain natriuretic peptide BNP (Log_10_BNP) and negatively with levels of hemoglobin and hematocrit, suggesting the influence of heart failure.

**Table 3:** Relationship Between p‐eNOS and Clinical Factors

No. of Patients	Basal p‐eNOS (69)		ΔINS (62)		ΔVEGF (30)		ΔACh (47)	
	ρ*/r*	*P* Value	ρ*/r*	*P* Value	ρ*/r*	*P* Value	ρ*/r*	*P* Value
Age, y	0.166	0.172	−0.170	0.187	0.062	0.746	−0.151	0.312
BMI, kg/m^2^	−0.175	0.149	0.145	0.260	−0.019	0.919	0.146	0.327
WBC, /μL	−0.042	0.734	0.167	0.193	−0.007	0.971	0.189	0.204
Hemoglobin, g/dL	−0.237	0.050*	0.167	0.195	0.110	0.562	0.005	0.975
Hematocrit, %	−0.294	0.014*	0.037	0.773	0.073	0.701	−0.012	0.936
Platelets, ×10^4^/μL	−0.131	0.282	0.045	0.726	0.167	0.377	0.090	0.547
AST, IU/L	0.076	0.535	−0.107	0.410	−0.447	0.013*	0.208	0.162
ALT, IU/L	−0.044	0.720	−0.075	0.563	−0.549	0.002*	0.050	0.740
LDL cholesterol, mg/dL	0.035	0.773	−0.013	0.198	0.250	0.182	−0.190	0.201
Triglyceride, mg/dL	−0.030	0.807	0.083	0.522	0.338	0.068	0.002	0.991
HDL cholesterol, mg/dL	−0.170	0.163	−0.067	0.604	0.063	0.740	−0.054	0.718
Glucose, mg/dL	−0.220	0.070	0.081	0.531	−0.109	0.568	−0.048	0.750
HbA1C, %	0.042	0.732	−0.100	0.439	0.000	0.998	−0.139	0.352
Uric acid, mg/dL	0.194	0.111	−0.005	0.967	−0.145	0.443	−0.144	0.336
eGFR, mL/min	−0.190	0.119	0.294	0.020*	0.196	0.298	0.345	0.018*
CRP, mg/dL	0.175	0.151	0.011	0.932	−0.064	0.735	−0.117	0.432
Log_10_BNP	0.288	0.016*	−0.266	0.037*	−0.305	0.101	−0.183	0.217
d‐ROMs (U.CARR.)†	0.094	0.475	−0.348	0.011*	−0.416	0.022*	−0.110	0.507
E/E′‡	0.039	0.778	−0.374	0.008*	−0.180	0.400	−0.191	0.245
DCT, ms§	0.065	0.626	−0.246	0.076	−0.331	0.091	−0.039	0.808
EF, %∥	−0.205	0.116	−0.076	0.581	−0.200	0.308	−0.060	0.703

ALT indicates alanine aminotransferase; AST, aspartate aminotransferase; BMI, body mass index; CRP, C‐reactive protein; DCT, deceleration time; d‐ROMs, derivatives of the reactive oxidative metabolites; E/E′, index of LV diastolic dysfunction; EF, ejection fraction; eGFR, estimated glomerular filtration rate; HbA1C, hemoglobin A1C; HDL, high‐density lipoprotein; LDL, low‐density lipoprotein; Log_10_BNP, log‐transformed brain natriuretic peptide (pg/mL); p‐eNOS, phosphorylated endogenous nitric oxide synthase at Ser1177; WBC, white blood cells; ΔACh, percentage change in ACh‐induced p‐eNOS; ΔINS, percentage change in insulin‐induced p‐eNOS; ΔVEGF, percentage change in VEGF‐induced p‐eNOS.

*p<0.05.

Numbers of basal p‐eNOS/ΔINS/ΔVEGF/ΔACh=^†^60/53/30/39, ^‡^54/49/24/39, ^§^58/53/27/42, ^∥^60/55/28/43.

### Clinical Factors Associated With eNOS Response to Insulin

**Figure 4:**
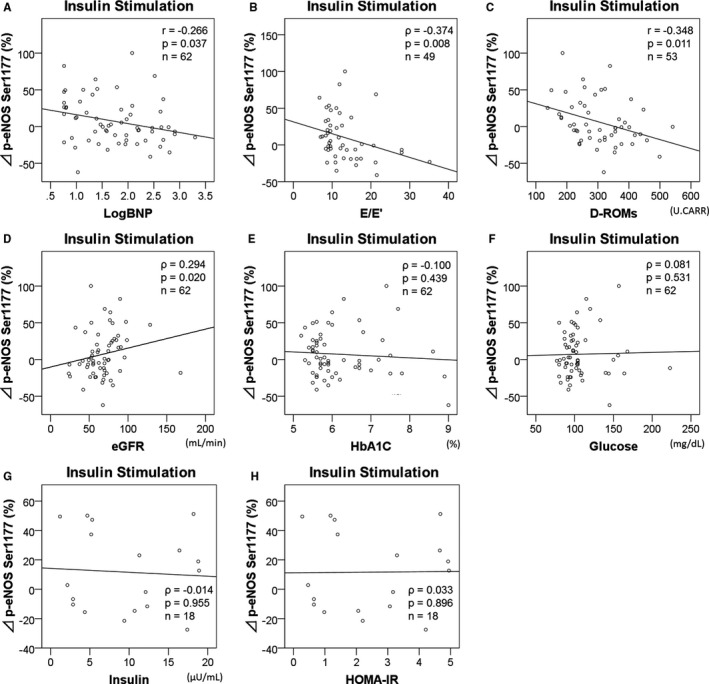
Correlations of insulin‐induced increase of p‐eNOS Ser1177 with confounding factors. Freshly isolated arterial endothelial cells were cultured in medium for 30 minutes before fixing with or without insulin (100 nmol/L, 30 minutes). The percentage increase in p‐eNOS by insulin from the basal p‐eNOS correlated with Log_10_ BNP (**A**), E/E′ (**B**), d‐ROMs (**C**) and eGFR (**D**), but did not correlate with HbA1C (**E**), fasting glucose level (**F**), fasting insulin level (**G**), HOMA‐IR (**H**). d‐ROMs indicates derivatives of the reactive oxidative metabolites; E/E′, index of LV diastolic dysfunction; eGFR, estimated glomerular filtration rate; HbA1C, hemoglobin A1C; HOMA‐IR, homeostatic model assessment of insulin resistance; Log_10_ BNP, log‐transformed brain natriuretic peptide (pg/mL); p‐eNOS, phosphorylated‐endogenous nitric oxide synthase at Ser1177.

The percentage change in insulin‐induced p‐eNOS (ΔINS, %) was higher in men than in women (men, 13±31, n=47; women, −12±25, n=15; *P*=0.005). ΔINS deteriorated with an increase in Log_10_BNP, E/E′ and d‐ROMs (Figure 4A through 4C) (Table 3). ΔINS was positively correlated with eGFR (Figure 4D). These results suggested an association with heart failure, oxidative stress and renal function.

In addition, ΔINS was not directly related to diabetes mellitus (DM), (DM, 8±40, n=20; others, 7±27, n=42; *P*=0.872). ΔINS did not correlate with HbA1C, fasting glucose level, fasting insulin level, homeostatic model assessment of insulin resistance (Figure 4E through 4H). However, ΔINS was significantly decreased in diabetics using self‐injections of insulin (insulin therapy, −29±18, n=5; others, 10±30, n=57; *P*=0.006). There were more women under insulin therapy than men (women, n=4/15 [27%]; men, n=1/47 [2%]; *P*=0.010). In a subgroup of type‐2 DM patients, ΔINS was significantly reduced in patients with CAD compared with those without it (CAD, −3±39, n=14; non‐CAD, 33±34, n=6; *P*=0.012).

### Clinical Factors Associated With eNOS Response to VEGF, and Acetylcholine

**Figure 5:**
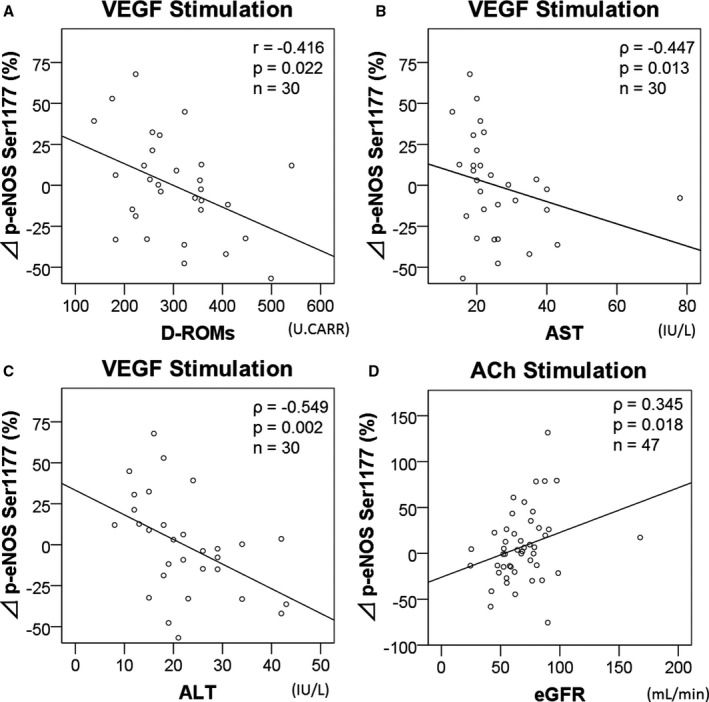
Correlations of VEGF, acetylcholine‐stimulated increase of p‐eNOS Ser1177 with confounding factors. Freshly acetylcholine arterial endothelial cells were cultured in medium with or without VEGF (20 ng/mL, 30 minutes), or acetylcholine (1 μmol/L, 15 minutes). The percentage increase in p‐eNOS by VEGF from the basal p‐eNOS correlated with d‐ROMs (**A**), AST (**B**) and ALT (**C**). The percentage increase of acetylcholine‐induced p‐eNOS correlated with eGFR (**D**). ALT indicates alanine aminotransferase; AST, aspartate aminotransferase; d‐ROMs, derivatives of the reactive oxidative metabolites; eGFR, estimated glomerular filtration rate; p‐eNOS, phospho‐endogenous nitric oxide synthase at Ser1177; VEGF, vascular endothelial growth factor.

The percentage change in VEGF‐induced p‐eNOS (ΔVEGF, %) was decreased in current smokers (current smoker, −16±22, n=12; others, 10±31, n=18; *P*=0.020). Furthermore, it was reduced in patients using furosemide (furosemide, −21±27, n=7; others, 6±29, n=23; *P*=0.037). ΔVEGF correlated negatively with d‐ROMs (Table 3, Figure 5A). Although ΔVEGF was not significantly associated with Log_10_BNP in this study, d‐ROMs were moderately associated with Log_10_BNP (*r*=0.516, n=60, *P*<0.001). ΔVEGF also correlated with serum levels of aspartate aminotransferase and alanine aminotransferase (Figure 5B and 5C).

In contrast, the percentage change in acetylcholine ‐induced p‐eNOS (ΔACh, %) increased with a decrease in eGFR (Figure 5D). ΔACh was higher in patients with chronic kidney disease defined as eGFR <60 mL/min (chronic kidney disease, −8±25, n=18; non‐chronic kidney disease, 16±43, n=29; *P*=0.037).

### Representative Images of eNOS Activation and CAVI

**Figure 6:**
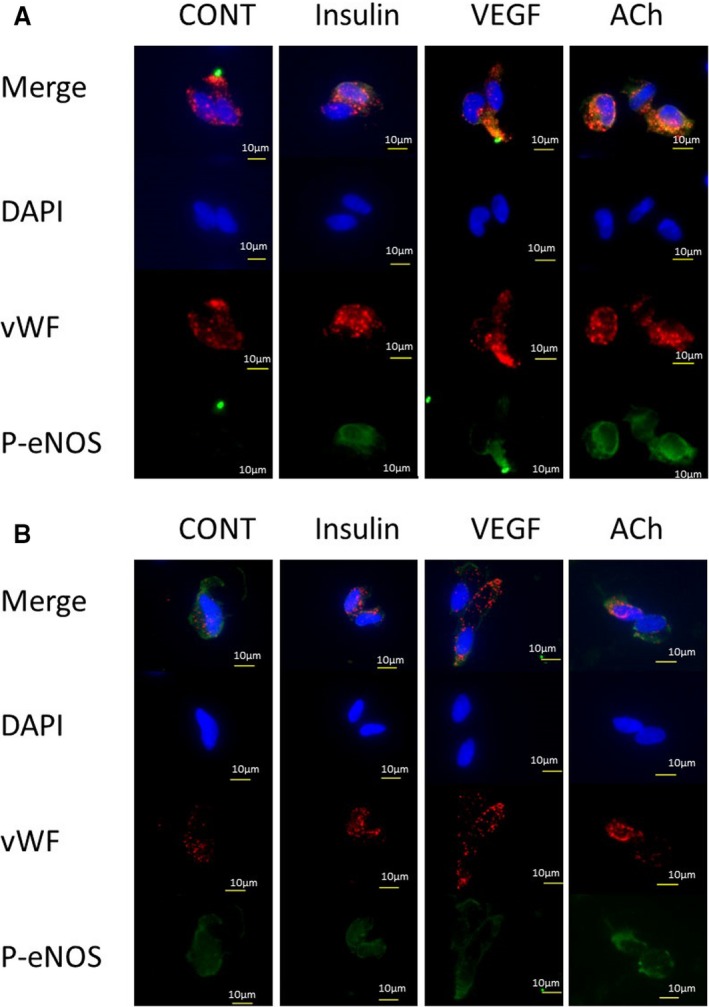
Images of p‐eNOS Ser1177 in patients with low and high CAVI scores. **A**, The images are from a 57‐year‐old non‐CAD man who had a history of smoking and obesity only (bone mass index: 27.1). CAVI was 7.77. Among laboratory data, BNP (25.4 pg/mL) and eGFR (97.4 mL/min) were within normal range. The basal p‐eNOS intensity was at low level (CONT). The intensity was increased by stimulation with insulin (+51%), VEGF (+45%), and acetylcholine (+79%). **B**, The images from a 75‐year‐old man with severe CAD and low cardiac function (ejection fraction 20%). He had diabetes mellitus, hypertension, hyperlipidemia, and was a current smoker. BNP was 1981.7 pg/mL (Log_10_ BNP: 3.30); however, renal function was within normal range (eGFR 69.8 mL/min). CAVI score was 10.11. The basal p‐eNOS was already enhanced (CONT), and the responses to stimuli were reduced. These resulted in decreases in the percentage changes induced by insulin (−1.6%) and VEGF (−27%). However, the acetylcholine‐induced p‐eNOS intensity was not reduced (+11%). Red—von Willebrand Factor; Green—p‐eNOS; Blue—DAPI. BMI indicates body mass index; BNP, brain natriuretic peptide (pg/mL); CAD, coronary artery disease; CAVI, cardio‐ankle vascular index; CONT, control; DAPI indicates 4′,6‐diamidino‐2‐phenylindole stain; eGFR, estimated glomerular filtration rate; p‐eNOS, phosphorylated endogenous nitric oxide synthase at Ser1177; VEGF, vascular endothelial growth factor; vWF, von Willebrand Factor.

The representative images of the 2 cases with high and low CAVI are shown in Figure 6. In the case of low CAVI, the basal p‐eNOS intensity was low while the insulin‐induced p‐eNOS intensity was high (Figure 6A). However, the difference was reduced in patients with high CAVI by both the elevation of basal intensity and reduced response to insulin (Figure 6B).

### Regression Analyses for CAVI

**Figure 7:**
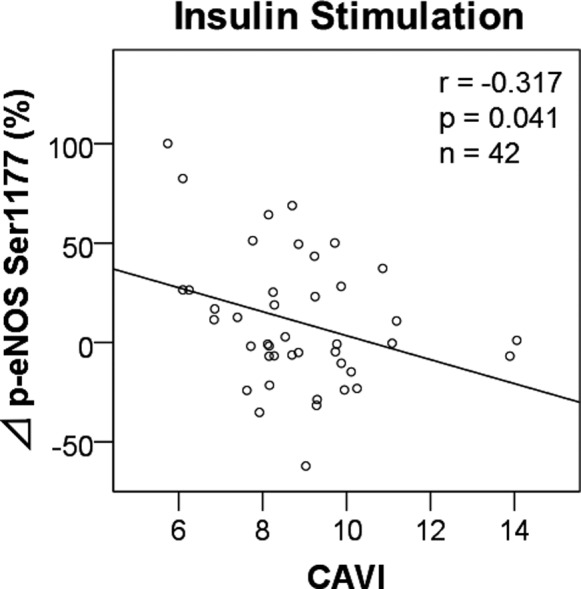
Correlations between p‐eNOS Ser1177 and CAVI. CAVI score correlated with percentage change of insulin‐induced p‐eNOS at Ser1177. CAVI indicates cardio‐ankle vascular index.

Univariate analysis showed that ΔINS was inversely associated with CAVI (Table 4, Figure 7). In multivariate regression analysis, the crude model (model 1) showed that the relationship between ΔINS and CAVI was not significant. However, the traditional stepwise regression model (model 2) and the adaptive Lasso regression model (model 3) showed that ΔINS was independently associated with CAVI (Figure S7).

**Table 4:** Regression Analysis for CAVI

No. of Patients	Univariate (46)		Multivariate (42)					
			Model 1		Model 2		Model 3	
	β	*P* Value	β	*P* Value	β	*P* Value	β	*P* Value
ΔINS, %	−0.317	0.041*, †	−0.291	0.059	−0.332	0.017*	−0.293	0.021*
Age, y	0.567	<0.001*	0.484	0.004*	0.475	<0.001*	0.489	<0.001*
Sex, man 1, woman 0	0.084	0.579	0.218	0.136	0.263	0.048*	0.213	0.025*
Hypertension, yes 1, no 0	0.098	0.516	0.046	0.757				
Hyperlipidemia, yes 1, no 0	0.127	0.400	−0.047	0.757				
Diabetes mellitus, yes 1, no 0	0.095	0.528	0.184	0.192			0.179	0.126
Current smoking, yes 1, no 0	−0.116	0.444	0.025	0.882				
Hemoglobin, g/dL	−0.376	0.010*	−0.048	0.862				
Hematocrit, %	−0.313	0.034*	−0.160	0.551	−0.255	0.038*	−0.209	0.010*
Platelets, ×10^4^/μL	−0.308	0.038*	−0.198	0.156			−0.171	0.099
eGFR, mL/min	−0.375	0.010*	−0.032	0.841				
d‐ROMs (U.CARR.)	0.331	0.034*, ‡						

This table displays the 3 models including ΔINS in multivariate regression model analysis. The adjusted *R* ^2^ was 0.405, *P*=0.003 for model 1; 0.509, *P*<0.001 for model 2; 0.560, *P*<0.001 for model 3, respectively. The original prediction formula and the solution path for model 3 were also shown in supplemental material. d‐ROMs indicates derivatives of the reactive oxidative metabolites; eGFR, estimated glomerular filtration rate; ΔINS, percentage change in insulin‐induced p‐eNOS at Ser1177.

*p<0.05.

†n=42.

‡n=41.

## Discussion

**Figure 8:**
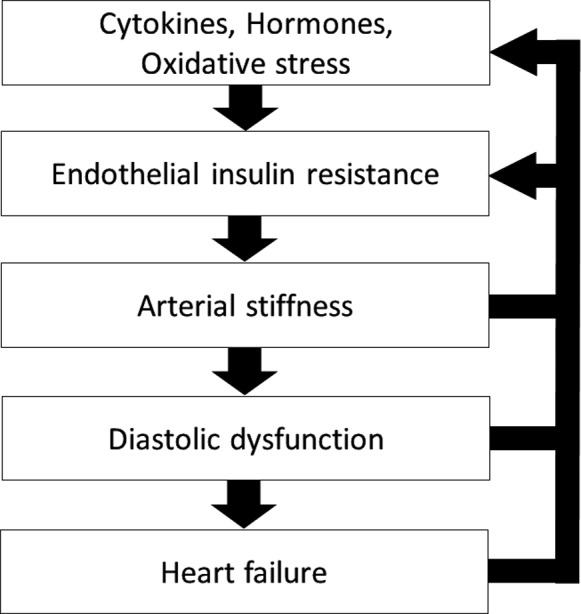
Overview of the relationship between endothelial insulin resistance and heart failure. The flowchart shows our view, which is that endothelial insulin resistance is associated with arterial stiffness and heart failure.

This cross‐sectional study using a novel method had 2 major findings. First, ΔINS inversely correlated with oxidative stress, Log_10_BNP and E/E′, an index of LV diastolic dysfunction on Doppler echocardiography. Second, ΔINS was independently associated with CAVI. These relationships are reasonable because CAVI is closely related to E/E′.^25^ Thus, endothelial IR was associated with arterial stiffness, LV diastolic dysfunction, and heart failure in this study. However, arterial stiffness and heart failure might induce endothelial IR (Figure 8).^26^, ^27^

Heart failure is a condition of high oxidative stress and the increased secretion of proinflammatory cytokines. Therefore, it is reasonable to think that signal transduction in the IRS‐1/PI3K/Akt/eNOS pathway could be impaired in heart failure.^26^ It was previously reported that insulin signaling can be impaired by non‐diabetic causes including oxidized low density lipoprotein cholesterol,^28^ angiotensin II,^29^, ^30^ vasopressin,^31^ TNF‐α,^32^ leptin,^33^, ^34^ uric acid,^35^ various hormones,^36^ and chronic kidney disease.^37^ Our results are concordant with clinical studies that have reported that endothelial dysfunction is accompanied with chronic congestive heart failure^38^, ^39^, ^40^ and increased mortality from heart failure.^41^

Arterial stiffness increases pulse wave reflection and causes LV diastolic dysfunction. Therefore, it is considered an exacerbating factor in heart failure.^42^, ^43^ The primary cause of arterial stiffness is a change in medial structural components.^44^ Matrix metalloproteinases promote overproduction of abnormal collagen and degradation of elastin in the extracellular matrix.^45^ Moreover, an accumulation of advanced glycation end products, calcium deposition, neurohormonal factors, and sympathetic nerve activation also play roles in development of arterial stiffness. In addition to the structural changes, endothelial dysfunction provokes vasomotor dysregulation and vascular stiffening.^46^, ^47^, ^48^ Therefore, endothelial IR, which indicates the dysregulation of IRS‐1/PI3‐kinase/Akt/eNOS signaling, could be involved in the pathogenesis of arterial stiffness.

Surprisingly, the patients with type‐2 DM in this study did not always have endothelial IR, although most previous studies have explored the mechanisms of endothelial IR in subjects with obesity or type‐2 DM. Because most patients had severe atherosclerotic disease and/or heart failure, the difference attributable to diabetes mellitus was difficult to be elucidated. However, all type‐2 DM patients under insulin therapy had decreased eNOS response to insulin. There were more women under insulin therapy than men. We think that was the main reason for the sex difference of ΔINS in our study. Additionally, a sub‐analysis of type‐2 DM patients revealed that insulin‐induced eNOS activation was significantly lower in diabetics who had CAD than those who did not. Thus, the duration, severity, and treatment of type‐2 DM might influence endothelial IR and further investigation is needed.

We tested the response of eNOS to stimuli other than insulin. VEGF is an important regulator of endothelial healing and growth and angiogenesis after vascular injury. VEGF mediates the release of NO via the IRS‐1/PI3‐kinase/Akt/eNOS pathway in the process.^49^ ΔVEGF was attenuated in smokers in our results. This is concordant with a previous study showing that cigarette smoke impairs VEGF‐dependent activation of the Akt/eNOS/NO pathway in human umbilical vein endothelial cells.^50^ Furthermore, ΔVEGF was associated with d‐ROMs similarly to ΔINS. We previously showed an association between d‐ROMs, serum levels of BNP, and high‐sensitivity C‐reactive protein.^51^ Because oxidative stress is a common mediator of atherosclerosis, heart failure, and inflammation, it is understandable that high‐oxidative stress can decrease ΔINS and ΔVEGF.

In contrast, the clinical factors associated with ΔACh did not match those of ΔINS and ΔVEGF. Various cascades in acetylcholine‐induced phosphorylation of eNOS have been previously reported.^52^, ^53^, ^54^, ^55^, ^56^ Acetylcholine induces rapid tyrosine phosphorylation and the activation of Janus kinase 2 signaling, which is upstream of the IRS‐1/PI 3‐kinase/Akt/eNOS pathway.^56^ This is an additional mechanism of acetylcholine‐induced eNOS activation transferred to a calcium‐dependent activation.^57^, ^58^ However, our sample size was too small to allow an evaluation of other signaling molecules underlying the activation of acetylcholine‐induced eNOS. Thus, further investigation is required to clarify the differences between ΔINS, ΔVEGF, and ΔACh.

To our knowledge, this is the first study to investigate freshly isolated arterial endothelial cells collected from a radial catheter sheath used for coronary angiography. Because commercialized human aortic endothelial cells and retrieved endothelial cells from animal tissues need stabilization in culture medium for a long time before experimental processes, we think that our non‐invasive method could be advantageous for evaluating the phenotypes of cardiovascular disease, including arterial stiffness. Furthermore, a future aim is to find a way to restore endothelial function and inhibit the progression of arterial stiffness. Many patients undergo repetitive coronary angiographies; thus this method is applicable for these patients so that the effects of drugs, nutrition, and lifestyle interventions can be seen.

This study had several limitations. First, the number of patients was too small to evaluate all underlying factors and drug effects that affect eNOS activity. The relatively small sample size for the CAVI analysis is concerning when attempting a multiple regression with a potentially large number of predictors. Therefore, we confirmed the association of ΔINS with CAVI by different modeling strategies. However, more samples are needed for accuracy. Second, this observational study did not have a control group of healthy participants because all participants had to have a reason to undergo coronary angiography. Third, we assessed endothelial IR with the percentage changes in p‐eNOS; however, total eNOS was not evaluated. There were possibilities that the variability of staining intensity could not be completely excluded even though control slides were used, and excessive standardization such as dividing p‐eNOS by total eNOS could diminish the accuracy. Accordingly, we thought that measurements of percentage change could be more reliable markers, because the effect of total eNOS and the variability of staining intensity were eliminated in the formula. In addition, the Western blots of the phosphorylated and total eNOS of the endothelial cells with or without insulin treatment are more reliable than immunofluorescence. Using some commercialized devices, Western blotting may be available from the limited number of the endothelial cells obtained from catheter sheaths. Thus, we need to confirm the results by multiple methods in the future.

## Conclusion

In conclusion, our findings suggested endothelial IR is associated with oxidative stress, LV diastolic dysfunction, arterial stiffness, and heart failure. This molecular biological assessment of freshly isolated arterial endothelial cells taken from radial sheaths may help us understand endothelial IR in clinical practice.

## Sources of Funding

This work was supported by a Grant‐in‐aid for Scientific Research from the Ministry of Education, Science, and Culture of Japan (to Masaki) (17K09565) and a grant (to Adachi) from the Ministry of Defense.

## Disclosures

None.

## Supporting information


**Data S1.** Supplemental experiments for quantification of immunofluorescent staining.
**Figure S1.** The validation of anti‐p‐eNOS antibody by western blotting.
**Figure S2.** The validation of anti‐p‐eNOS antibody by immunohistochemistry.
**Figure S3.** Optimal timing for evaluating immunofluorescent intensity.
**Figure S4.** The relationship between intensities of western blotting and immunofluorescence of human umbilical vein endothelial cells after insulin‐stimulation.
**Figure S5.** Elimination of p‐eNOS Ser1177 by eNOS knockdown.
**Figure S6.** Augmentation of p‐eNOS Ser1177 by serum‐stimulation.
**Figure S7.** Solution path.

## References

[1] Riehle C , Abel ED . Insulin signaling and heart failure. Circ Res. 2016;118:1151–1169. 27034277 10.1161/CIRCRESAHA.116.306206 PMC4833475

[2] Fulton DJR . Mechanisms of vascular insulin resistance. A substitute Akt? Circ Res. 2009;104:1035–1037. 19423861 10.1161/CIRCRESAHA.109.198028 PMC2974618

[3] Muniyappa R , Sowers JR . Role of insulin resistance in endothelial dysfunction. Rev Endocr Metab Disord. 2013;14:5–12. 23306778 10.1007/s11154-012-9229-1 PMC3594115

[4] Fulton D , Gratton JP , McCabe TJ , Fontana J , Fujio Y , Walsh K , Franke TF , Papapetropoulos A , Sessa WC . Regulation of endothelium‐derived nitric oxide production by the protein kinase Akt. Nature. 1999;399:597–601. 10376602 10.1038/21218 PMC3637917

[5] Montagnani M , Chen H , Barr VA , Quon MJ . Insulin‐stimulated activation of eNOS is independent of Ca^2+^ but requires phosphorylation by Akt at Ser(1179). J Biol Chem. 2001;276:30392–30398. 11402048 10.1074/jbc.M103702200

[6] Zhang QJ , McMillin SL , Tanner JM , Palionyte M , Abel ED , Symons JD . Endothelial nitric oxide synthase phosphorylation in treadmill‐running mice: role of vascular signalling kinases. J Physiol. 2009;587:3911–3920. 19505983 10.1113/jphysiol.2009.172916 PMC2746618

[7] Gélinas DS , Bernatchez PN , Rollin S , Bazan NG , Sirois MG . Immediate and delayed VEGF‐mediated NO synthesis in endothelial cells: role of PI3K, PKC and PLC pathways. Br J Pharmacol. 2002;137:1021–1030. 12429574 10.1038/sj.bjp.0704956 PMC1573579

[8] Carreau A , Kieda C , Grillon C . Nitric oxide modulates the expression of endothelial cell adhesion molecules involved in angiogenesis and leukocyte recruitment. Exp Cell Res. 2011;317:29–41. 20813110 10.1016/j.yexcr.2010.08.011

[9] Apostoli GL , Solomon A , Smallwood MJ , Winyard PG , Emerson M . Role of inorganic nitrate and nitrite in driving nitric oxide‐cGMP‐mediated inhibition of platelet aggregation in vitro and in vivo. J Thromb Haemost. 2014;12:1880–1889. 25163536 10.1111/jth.12711

[10] Qiu J , Zheng Y , Hu J , Liao D , Gregersen H , Deng X , Fan Y , Wang G . Biomechanical regulation of vascular smooth muscle cell functions: from in vitro to in vivo understanding. J R Soc Interface. 2013;11:20130852. 24152813 10.1098/rsif.2013.0852 PMC3836323

[11] Tabit CE , Shenouda SM , Holbrook M , Fetterman JL , Kiani S , Frame AA , Kluge MA , Held A , Dohadwala MM , Gokce N , Farb MG , Rosenzweig J , Ruderman N , Vita JA , Hamburg NM . Protein kinase C‐β contributes to impaired endothelial insulin signaling in humans with diabetes mellitus. Circulation. 2013;127:86–95. 23204109 10.1161/CIRCULATIONAHA.112.127514 PMC3572725

[12] Bretón‐Romero R , Feng B , Holbrook M , Farb MG , Fetterman JL , Linder EA , Berk BD , Masaki N , Weisbrod RM , Inagaki E , Gokce N , Fuster JJ , Walsh K , Hamburg NM . Endothelial dysfunction in human diabetes is mediated by Wnt5a‐JNK signaling. Arterioscler Thromb Vasc Biol. 2016;36:561–569. 26800561 10.1161/ATVBAHA.115.306578 PMC4913891

[13] Spoto B , Pisano A , Zoccali C . Insulin resistance in chronic kidney disease: a systematic review. Am J Physiol Renal Physiol. 2016;311:F1087–F1108. 27707707 10.1152/ajprenal.00340.2016

[14] Shirai K , Hiruta N , Song M , Kurosu T , Suzuki J , Tomaru T , Miyashita Y , Saiki A , Takahashi M , Suzuki K , Takata M . Cardio‐ankle vascular index (CAVI) as a novel indicator of arterial stiffness: theory, evidence and perspectives. J Atheroscler Thromb. 2011;18:924–938. 21628839 10.5551/jat.7716

[15] Imai E , Horio M , Nitta K , Yamagata K , Iseki K , Hara S , Ura N , Kiyohara Y , Hirakata H , Watanabe T , Moriyama T , Ando Y , Inaguma D , Narita I , Iso H , Wakai K , Yasuda Y , Tsukamoto Y , Ito S , Makino H , Hishida A , Matsuo S . Estimation of glomerular filtration rate by the MDRD study equitation modified for Japanese patients with chronic kidney disease. Clin Exp Nephrol. 2006;11:41–50. 10.1007/s10157-006-0453-4 17384997

[16] Alberti A , Bolognini L , Macciantelli D , Caratelli M . The radical cation of N, N‐diethylpara‐phenylendiamine: a possible indicator of oxidative stress in biological samples. Res Chem Intermed. 2000;26:253–267.

[17] Cesarone MR , Belcaro G , Carratelli M , Cornelli U , De Sanctis MT , Incandela L , Barsotti A , Terranova R , Nicolaides A . A simple test to monitor oxidative stress. Int Angiol. 1999;18:127–130. 10424368

[18] Haffner SM , Miettinen H , Stern MP . The homeostasis model in the San Antonio Heart Study. Diabetes Care. 1997;20:1087–1092. 9203442 10.2337/diacare.20.7.1087

[19] Bonarjee VVS . Arterial stiffness: a prognostic marker in coronary heart disease. available methods and clinical application. Front Cardiovasc Med. 2018;5:64. 29951487 10.3389/fcvm.2018.00064 PMC6008540

[20] Gohbara M , Iwahashi N , Sano Y , Akiyama E , Maejima N , Tsukahara K , Hibi K , Kosuge M , Ebina T , Umemura S , Kimura K . Clinical impact of the cardio‐ankle vascular index for predicting cardiovascular events after acute coronary syndrome. Circ J. 2016;80:1420–1426. 27116899 10.1253/circj.CJ-15-1257

[21] Harrell F . Regression Modeling Strategies. Cham, Switzerland: Springer; 2015:63–102.

[22] Thors B , Halldórsson H , Jónsdóttir G , Thorgeirsson G . Mechanism of thrombin mediated eNOS phosphorylation in endothelial cells is dependent on ATP levels after stimulation. Biochim Biophys Acta. 2008;1783:1893–1902. 18687367 10.1016/j.bbamcr.2008.07.003

[23] Motley ED , Eguchi K , Patterson MM , Palmer PD , Suzuki H , Eguchi S . Mechanism of endothelial nitric oxide synthase phosphorylation and activation by thrombin. Hypertension. 2007;49:577–583. 17210830 10.1161/01.HYP.0000255954.80025.34

[24] Touyz RM . Regulation of endothelial nitric oxide synthase by thrombin. Hypertension. 2007;49:429–431. 17210837 10.1161/01.HYP.0000255955.75119.1a

[25] Namba T , Masaki N , Matsuo Y , Sato A , Kimura T , Horii S , Yasuda R , Yada H , Kawamura A , Takase B , Adachi T . Arterial stiffness is significantly associated with left ventricular diastolic dysfunction in patients with cardiovascular disease. Int Heart J. 2016;57:729–735. 27829641 10.1536/ihj.16-112

[26] Aroor AR , Mandavia CH , Sowers JR . Insulin resistance and heart failure: molecular mechanisms. Heart Fail Clin. 2012;8:609–617. 22999243 10.1016/j.hfc.2012.06.005 PMC3457065

[27] Mozos I , Malainer C , Horbańczuk J , Gug C , Stoian D , Luca CT , Atanasov AG . Inflammatory markers for arterial stiffness in cardiovascular diseases. Front Immunol. 2017;8:1058. 28912780 10.3389/fimmu.2017.01058 PMC5583158

[28] Clavreul N , Bachschmid MM , Hou X , Shi C , Idrizovic A , Ido Y , Pimentel D , Cohen RA . S‐glutathiolation of p21ras by peroxynitrite mediates endothelial insulin resistance caused by oxidized low‐density lipoprotein. Arterioscler Thromb Vasc Biol. 2006;26:2454–2461. 16931794 10.1161/01.ATV.0000242791.28953.4c

[29] Andreozzi F , Laratta E , Sciacqua A , Perticone F , Sesti G . Angiotensin II impairs the insulin signaling pathway promoting production of nitric oxide by inducing phosphorylation of insulin receptor substrate‐1 on Ser312 and Ser616 in human umbilical vein endothelial cells. Circ Res. 2004;94:1211–1218. 15044323 10.1161/01.RES.0000126501.34994.96

[30] Aroor AR , Demarco VG , Jia G , Sun Z , Nistala R , Meininger GA , Sowers JR . The role of tissue renin‐angiotensin‐aldosterone system in the development of endothelial dysfunction and arterial stiffness. Front Endocrinol (Lausanne). 2013;4:161. 24194732 10.3389/fendo.2013.00161 PMC3810594

[31] Kaufmann JE , Iezzi M , Vischer UM . Desmopressin (DDAVP) induces NO production in human endothelial cells via V2 receptor‐ and cAMP‐mediated signaling. J Thromb Haemost. 2003;1:821–828. 12871421 10.1046/j.1538-7836.2003.00197.x

[32] da Costa RM , Neves KB , Mestriner FL , Louzada‐Junior P , Bruder‐Nascimento T , Tostes RC . TNF‐α induces vascular insulin resistance via positive modulation of PTEN and decreased Akt/eNOS/NO signaling in high fat diet‐fed mice. Cardiovasc Diabetol. 2016;15:119. 27562094 10.1186/s12933-016-0443-0 PMC5000486

[33] Ding N , Liu B , Song J , Bao S , Zhen J , Lv Z , Wang R . Leptin promotes endothelial dysfunction in chronic kidney disease through AKT/GSK3β and β‐catenin signals. Biochem Biophys Res Commun. 2016;480:544–551. 27789284 10.1016/j.bbrc.2016.10.079

[34] Vecchione C , Maffei A , Colella S , Aretini A , Poulet R , Frati G , Gentile MT , Fratta L , Trimarco V , Trimarco B , Lembo G . Leptin effect on endothelial nitric oxide is mediated through Akt‐endothelial nitric oxide synthase phosphorylation pathway. Diabetes. 2002;51:168–173. 11756337 10.2337/diabetes.51.1.168

[35] Choi YJ , Yoon Y , Lee KY , Hien TT , Kang KW , Kim KC , Lee J , Lee MY , Lee SM , Kang DH , Lee BH . Uric acid induces endothelial dysfunction by vascular insulin resistance associated with the impairment of nitric oxide synthesis. FASEB J. 2014;28:3197–3204. 24652948 10.1096/fj.13-247148

[36] Duckles Sue P . Hormonal modulation of endothelial NO production. Pflugers Arch. 2010;459:841–851. 20213497 10.1007/s00424-010-0797-1 PMC2865573

[37] Zhou QG , Fu XJ , Xu GY , Cao W , Liu HF , Nie J , Liang M , Hou FF . Vascular insulin resistance related to endoplasmic reticulum stress in aortas from a rat model of chronic kidney disease. Am J Physiol Heart Circ Physiol. 2012;303:H1154–H1165. 22942179 10.1152/ajpheart.00407.2012

[38] Katz SD , Biasucci L , Sabba C , Strom JA , Jondeau G , Galvao M , Solomon S , Nikolic SD , Forman R , LeJemtel TH . Impaired endothelium‐mediated vasodilation in the peripheral vasculature of patients with congestive heart failure. J Am Coll Cardiol. 1992;19:918–925. 1552112 10.1016/0735-1097(92)90271-n

[39] Drexler H , Hayoz D , Munzel T , Hornig B , Just H , Brunner HR , Zelis R . Endothelial function in chronic congestive heart failure. Am J Cardiol. 1992;69:1596–1601. 1598876 10.1016/0002-9149(92)90710-g

[40] Marti CN , Gheorghiade M , Kalogeropoulos AP , Georgiopoulou VV , Quyyumi AA , Butler J . Endothelial dysfunction, arterial stiffness, and heart failure. J Am Coll Cardiol. 2012;60:1455–1469. 22999723 10.1016/j.jacc.2011.11.082

[41] Katz SD , Hryniewicz K , Hriljac I , Balidemaj K , Dimayuga C , Hudaihed A , Yasskiy A . Vascular endothelial dysfunction and mortality risk in patients with chronic heart failure. Circulation. 2005;111:310–314. 15655134 10.1161/01.CIR.0000153349.77489.CF

[42] Lee HY , Oh BH . Aging and arterial stiffness. Circ J. 2010;74:2257–2262. 20962429 10.1253/circj.cj-10-0910

[43] Chow B , Rabkin SW . The relationship between arterial stiffness and heart failure with preserved ejection fraction: a systemic meta‐analysis. Heart Fail Rev. 2015;20:291–303. 25716909 10.1007/s10741-015-9471-1

[44] Zieman SJ , Melenovsky V , Kass DA . Mechanisms, pathophysiology, and therapy of arterial stiffness. Arterioscler Thromb Vasc Biol. 2005;25:932–943. 15731494 10.1161/01.ATV.0000160548.78317.29

[45] Yasmin , Wallace S , McEniery CM , Dakham Z , Pusalkar P , Maki‐Petaja K , Ashby MJ , Cockcroft JR , Wilkinson IB . Matrix metalloproteinase‐9 (MMP‐9), MMP‐2, and serum elastase activity are associated with systolic hypertension and arterial stiffness. Arterioscler Thromb Vasc Biol. 2005;25:372–378. 15556929 10.1161/01.ATV.0000151373.33830.41

[46] Wilkinson IB , Franklin SS , Cockcroft JR . Nitric oxide and the regulation of large artery stiffness: from physiology to pharmacology. Hypertension. 2004;44:112–116. 15262901 10.1161/01.HYP.0000138068.03893.40

[47] McEniery CM , Wallace S , Mackenzie IS , McDonnell B , Yasmin Newby DE , Cockcroft JR , Wilkinson IB . Endothelial function is associated with pulse pressure, pulse wave velocity, and augmentation index in healthy humans. Hypertension. 2006;48:602–608. 16940223 10.1161/01.HYP.0000239206.64270.5f

[48] Brandes RP , Fleming I , Busse R . Endothelial aging. Cardiovasc Res. 2005;66:286–294. 15820197 10.1016/j.cardiores.2004.12.027

[49] Abeyrathna P , Su Y . The critical role of Akt in cardiovascular function. Vascul Pharmacol. 2015;74:38–48. 26025205 10.1016/j.vph.2015.05.008 PMC4659756

[50] Michaud SE , Dussault S , Groleau J , Haddad P , Rivard A . Cigarette smoke exposure impairs VEGF‐induced endothelial cell migration: role of NO and reactive oxygen species. J Mol Cell Cardiol. 2006;41:275–284. 16806264 10.1016/j.yjmcc.2006.05.004

[51] Masaki N , Sato A , Horii S , Kimura T , Toya T , Yasuda R , Namba T , Yada H , Kawamura A , Adachi T . Usefulness of the d‐ROMs test for prediction of cardiovascular events. Int J Cardiol. 2016;222:226–232. 27497099 10.1016/j.ijcard.2016.07.225

[52] Kobayashi T , Nemoto S , Ishida K , Taguchi K , Matsumoto T , Kamata K . Involvement of CaM kinase II in the impairment of endothelial function and eNOS activity in aortas of Type 2 diabetic rats. Clin Sci. 2012;123:375–386. 22494112 10.1042/CS20110621

[53] Murthy S , Koval OM , Ramiro Diaz JM , Kumar S , Nuno D , Scott JA , Allamargot C , Zhu LJ , Broadhurst K , Santhana V , Kutschke WJ , Irani K , Lamping KG , Grumbach IM . Endothelial CaMKII as a regulator of eNOS activity and NO‐mediated vasoreactivity. PLoS One. 2017;12:e0186311. 29059213 10.1371/journal.pone.0186311 PMC5653296

[54] Adapala RK , Talasila PK , Bratz IN , Zhang DX , Suzuki M , Meszaros JG , Thodeti CK . PKCα mediates acetylcholine induced activation of TRPV4‐dependent calcium influx in endothelial cells. Am J Physiol Heart Circ Physiol. 2011;301:H757–H765. 21705673 10.1152/ajpheart.00142.2011 PMC3302191

[55] Kitayama J , Kitazono T , Ibayashi S , Wakisaka M , Watanabe Y , Kamouchi M , Nagao T , Fujishima M . Role of phosphatidylinositol 3‐kinase in acetylcholine‐induced dilatation of rat basilar artery. Stroke. 2000;31:2487–2493. 11022083 10.1161/01.str.31.10.2487

[56] Zecchin HG , Priviero FB , Souza CT , Zecchin KG , Prada PO , Carvalheira JB , Velloso LA , Antunes E , Saad MJ . Defective insulin and acetylcholine induction of endothelial cell‐nitric oxide synthase through insulin receptor substrate/Akt signaling pathway in aorta of obese rats. Diabetes. 2007;56:1014–1024. 17229938 10.2337/db05-1147

[57] Dudzinski DM , Michel T . Life history of eNOS: partners and pathways. Cardiovasc Res. 2007;75:247–260. 17466957 10.1016/j.cardiores.2007.03.023 PMC2682334

[58] Balligand JL , Feron O , Dessy C . eNOS activation by physical forces: from short‐term regulation of contraction to chronic remodeling of cardiovascular tissues. Physiol Rev. 2009;89:481–534. 19342613 10.1152/physrev.00042.2007

