# Supplementary material for: Endothelial Insulin Resistance of Freshly Isolated Arterial Endothelial Cells From Radial Sheaths in Patients With Suspected Coronary Artery Disease
Source: J Am Heart Assoc. 2019 Mar 19;8(6):e010816. doi: 10.1161/JAHA.118.010816 (PMC6475050; doi:10.1161/JAHA.118.010816)
Supplement: Supplementary file 1 — Data S1. Supplemental experiments for quantification of immunofluorescent staining. Figure S1. The validation of anti‐p‐eNOS antibody by western blotting. Figure S2. The validation of anti‐p‐eNOS antibody by immunohistochemistry. Figure S3. Optimal timing for evaluating immunofluorescent intensity. Figure S4. The relationship between intensities of western blotting and immunofluorescence of human umbilical vein endothelial cells after insulin‐stimulation. Figure S5. Elimination of p‐eNOS Ser1177 by eNOS knockdown. Figure S6. Augmentation of p‐eNOS Ser1177 by serum‐stimulation. Figure S7. Solution path. [file JAH3-8-e010816-s001.pdf]

SUPPLEMENTAL MATERIAL

Data S1.

Supplemental Introduction

The experimental method was referring to our previous studies,[1,2] in which freshly isolated endothelial cells were obtained by wire-abrasion of upper-arm vein. However, this is the first study to use endothelial cells collected from radial catheter sheath. The non-invasive assessment enables us to measure and compare endothelial insulin resistance in many patients. Therefore, 1) validation of anti-phospho-eNOS antibody, and 2) optimization of quantifying immunofluorescence intensity should be reconfirmed. Furthermore, positive control and negative control were encouraged for validating the quantification of immunofluorescence intensity. For the reasons, we added the following data, which support the effectiveness of our method and the results.

In addition, we attached the solution path of the adaptive Lasso model to show the process of selecting the independent factors for the cardio-ankle vascular index (CAVI).

Supplemental Methods

Endothelial cells

Human umbilical vein endothelial cells (HUVECs) of passage 3-4 were cultured in dish or 4-well chamber slides. The cells were starved for 24 hours with serum-free medium. The cells were collected with lysate buffer for western blotting or fixed with 4% paraformaldehyde for immunofluorescence microscopy.

Western blot analysis

Proteins were subjected to 4 to 12% gradient gels (Invitrogen) and transferred to polyvinylidene difluoride membranes. Membranes were initially blocked (Blocking one, Nacalai Tesque, Kyoto, Japan) for 1 hour. Membranes were cut and probed in blocking buffer containing primary antibodies of 1:2000 dilution: phosphorylated eNOS at serine 1177 (GeneTex, Irvine, CA), followed by the appropriate horseradish peroxidase–conjugated secondary antibody. Immunoreactions were visualized with SuperSignal® West Dura Extended Duration Substrate (Thermo Scientific). Membranes were stripped (WB Stripping Solution, Nacalai Tesque) for 30 minutes at room temperature were

probed with phosphorylated eNOS at serine 1177 (1:1000 dilution; Cell Signaling, Danvers, MA), eNOS/NOS Type III (1:2000 dilution; BD Biosciences, San Jose, CA). The other cuts of membranes were probed with phosphorylated Akt at serine 473, total Akt [1:1000 dilution; Cell Signaling (CST)], GAPDH antibodies of 1:1000 dilution to verify equal protein loading. The bands were quantified by densitometry.

Immunohistochemistry

The method was described in our previous paper.[3] Briefly, formalin-fixed saphenous veins were embedded in paraffin, and washed with xylene, 100% ethanol, 95% ethanol, and water for slide deparaffinization. Endogenous peroxidase was removed by treatment with 3% H_2O_2 for 5 minutes and washing it under running water for 5 minutes. Antigen retrieval was performed by using the autoclave method (20 minutes at 121°C) and a citrate buffer (10 mmol/L, pH 8). After cooling, the plate was washed with phosphate-buffered saline (1.37 mol/L NaCl, 27 mmol/L KCl, 81 mmol/L Na_2HPO_4, 12 H_2O, 14.7 mmol/L KH_2PO_4) and blocking was performed for 60 minutes using Blocking One (1/5, Nacalai Tesque). Incubation was performed overnight at 4°C with the primary

antibody (p-eNOS Ser1177, 1:50, GTX50212, GeneTex). After washing with phosphate-buffered saline, the secondary antibody (Histofine R Simple Stain MAX PO MULTI, Nichirei Bioscience Inc.) was reacted for 1 h. Finally, 3,3'-diaminobenzidine was added and allowed to react for 10 minutes, before the samples were dehydrated, penetrated, and re-sealed.

Assessment of protein expression by quantitative Immunofluorescence

Fixed sample slides were thawed and rehydrated with PBS containing 50 mmol/L glycine (Sigma) for 10 minutes. The cells on the slides were permeabilized with 0.1% Triton X-100, and nonspecific binding sites were blocked with 0.5% BSA. The slides were incubated overnight at 4°C with primary antibodies against the following targets: p-eNOS Ser1177 (1:200 dilution; GeneTex). All of the slides were double-stained with an anti–von Willebrand Factor (vWF) antibody (1:300 dilution; Invitrogen, Carlsbad, CA), or eNOS/NOS Type III (1:200 dilution; BD Biosciences) for identification of endothelial cell. After the incubation, the slides were washed and incubated for 1 hour at 37°C with corresponding Alexa Fluor-488 and Alexa Fluor-594 antibodies (1:200 dilution; Invitrogen). The slides were washed again and

mounted under glass coverslips with Vectashield containing DAPI for nuclear identification (Vector Laboratories, Burlingame, CA).

Slide images of a fluorescence microscope at × 20 magnification were captured (KEYENCE, Osaka, Japan). Exposure time was constant, and image intensity was corrected for background fluorescence. Fluorescent intensity was quantified by a software (KEYENCE, Osaka, Japan). For each protein of interest, fluorescent intensity was quantified in 20 cells from each slide and averaged.

Making lentivirus vector expressing short-hairpin RNA to knock-down eNOS.

The target sequence (GGAACAGCACAAGAGTTA) was designed from Human NOS3 mRNA sequence (NM_000603.4). This sequence recognizes all isoforms of NOS3 and substantially (5 nt) differs from potential off-target sequences by Blast analysis. ShRNA expressing lentivirus was made as published by us before.[4]

Supplemental Results

Validation of anti-phospho-eNOS antibody used in the study

The method of this study is highly dependent on the quality of antibody. The western blot with anti-p-eNOS Ser1177 antibody (GTX50212, GeneTex), which were used for immunofluorescence in the study, exhibits a single band just below 150 kD (Figure S1A). The same membrane was stained with anti-p-eNOS Ser1177 antibody (CST #9571) (Figure S1B) and subsequently with anti-total-eNOS antibody (BD 610297) (Figure S1C). The bands located same molecular weight. Therefore, we confirmed that changes of immunofluorescent intensity come from the protein of this band. Thus, the anti-p-eNOS antibody (GTX50212) is available for quantification of immunofluorescent intensity.

Figure S2 shows the sections of a human saphenous vein harvested from a same patient during coronary artery bypass grafting. The intima was stained by anti-p-eNOS antibody (GTX50212) (left) as same as anti-total-eNOS antibody (BD 610297) (right). The image of vascular endothelial growth factor (VEGF)-stimulated section is also available in our recent paper.[3] The intensity was thickened by 30 minutes stimulation of VEGF.

Optimization of quantifying immunofluorescence intensity

HUVECs were cultured in 4-well chamber slides (Figure S3A). The cells were starved for 24 hours with serum-free medium and fixed with 4% paraformaldehyde at each time point after stimulation. From the results, the timing of evaluating immunofluorescence intensity was considered appropriate at 30 minutes for insulin, VEGF, and 15 minutes for ACh (Figure S3B).

Figure S4A shows the immunoblotting of the HUVECs with p-eNOS Ser1177 (GTX50212). eNOS was activated after increase of p-Akt Ser473 (CST #4060). The comparison between western blotting and immunofluorescence is shown in Figure S4B. The inter-class correlation was 0.928 ($p = 0.004$). The result indicates that the quantification of immunofluorescent intensity is functional to detect the change of p-eNOS Ser1177.

Validation of method with eNOS knockdown HUVECs

We created HUVECs with eNOS knock-down (KD) for negative controls to test our assessment of immunofluorescent microscopy. The images of HUVECs after insulin stimulation treated by KD were shown in Figure S5A. The total

eNOS (red) was reduced by KD. The p-eNOS Ser1177 (GTX50212, green) was extinguished regardless of insulin stimulation.

Figure S5B shows the western blotting of HUVECs with or without KD. The bands of p-eNOS Ser1177 (GTX50212) was eliminated by KD. Thus, these results reconfirmed the validation of the antibody.

Positive control with serum-stimulation

It was previously known that thrombin increases eNOS activation.[5-7] Figure S6A shows the increased immunofluorescent intensity of p-eNOS Ser1177 by culturing with human fresh serum for 30 minutes before fixation. The effect was not seen in the HUVECs treated by KD. The western blotting confirmed that p-eNOS Ser1177 increased by serum after augmentation of p-Akt Ser473 (Figure S6B)

. This serum-stimulated increase of p-eNOS Ser1177 was absent by KD (Figure S6C) as same as other stimulations (Figure S6D). Therefore, we applied serum-stimulation to freshly isolated arterial endothelial cells as a positive control. The results were described in the main text (Figure 3).

The supplemental data of the adaptive Lasso regression for CAVI.

Figure S7 shows the solution path of the adaptive Lasso regression model (model 3 in Table 4) described by JMP pro. version 13.1.0 (SAS Institute Japan, Tokyo).

Figure S1. The validation of anti-p-eNOS antibody by western blotting.

A

P-eNOS Ser1177 (GTX50212)

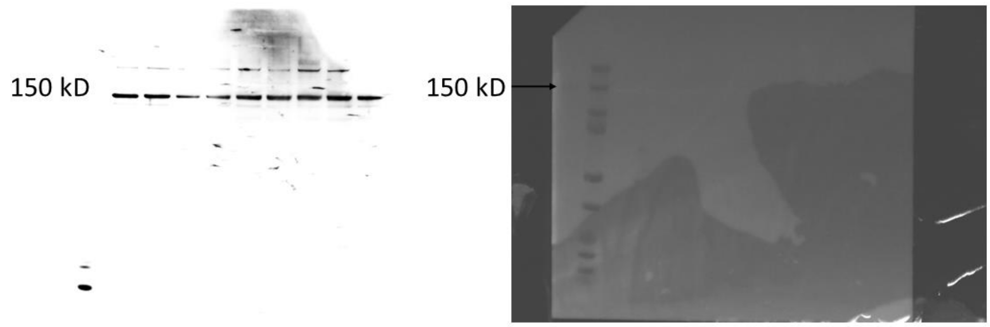

B

P-eNOS Ser1177 (CST 9571)

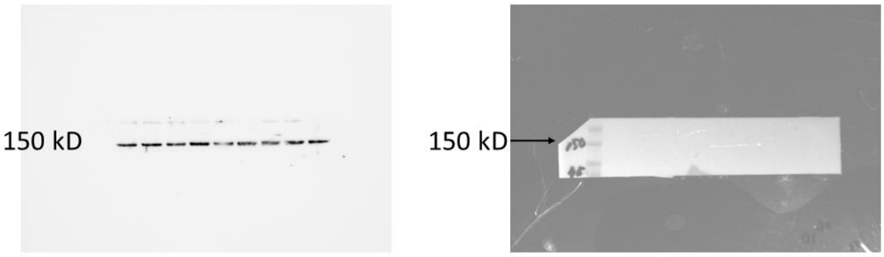

C

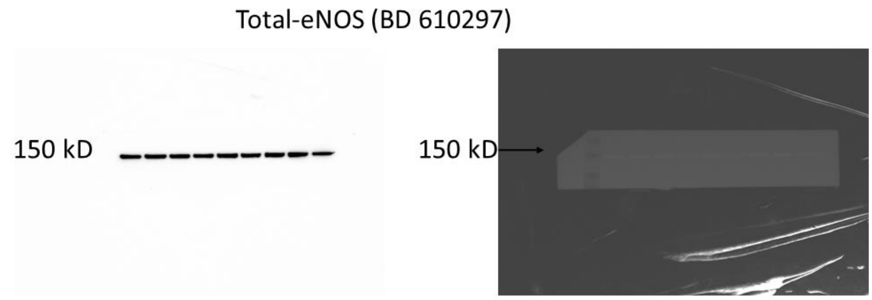

A: The western blot with anti-p-eNOS Ser1177 antibody (GTX50212), B: anti-p-eNOS Ser1177 antibody (CST #9571), C: anti-total-eNOS antibody (BD 610297). The figures are a chemifluorescent image (left) and a digitizing image (right).

Figure S2. The validation of anti-p-eNOS antibody by immunohistochemistry.

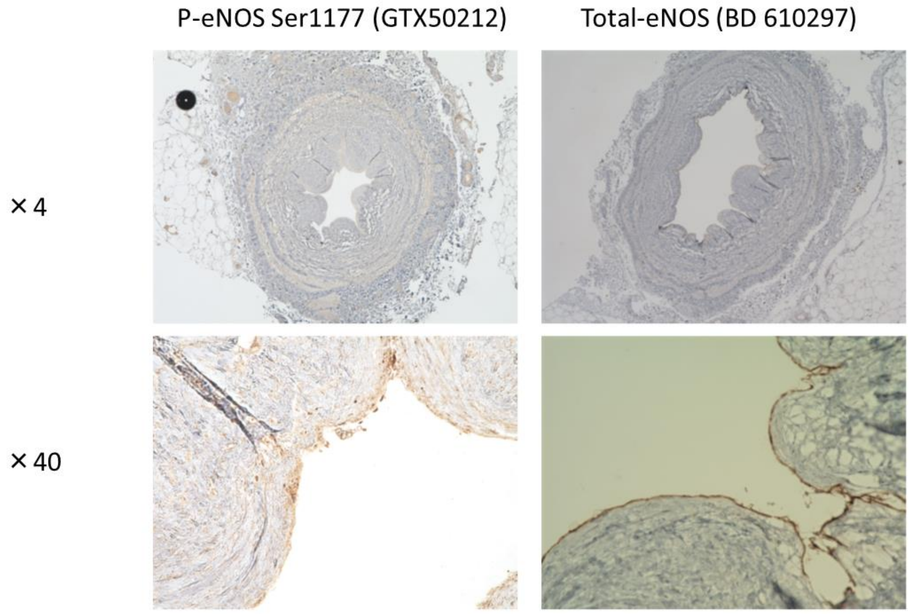

The images show the location probed by the anti-p-eNOS Ser1177 antibody (GTX50212) (left) and anti-total-eNOS antibody (BD 610297) (right).

Figure S3. Optimal timing for evaluating immunofluorescent intensity.

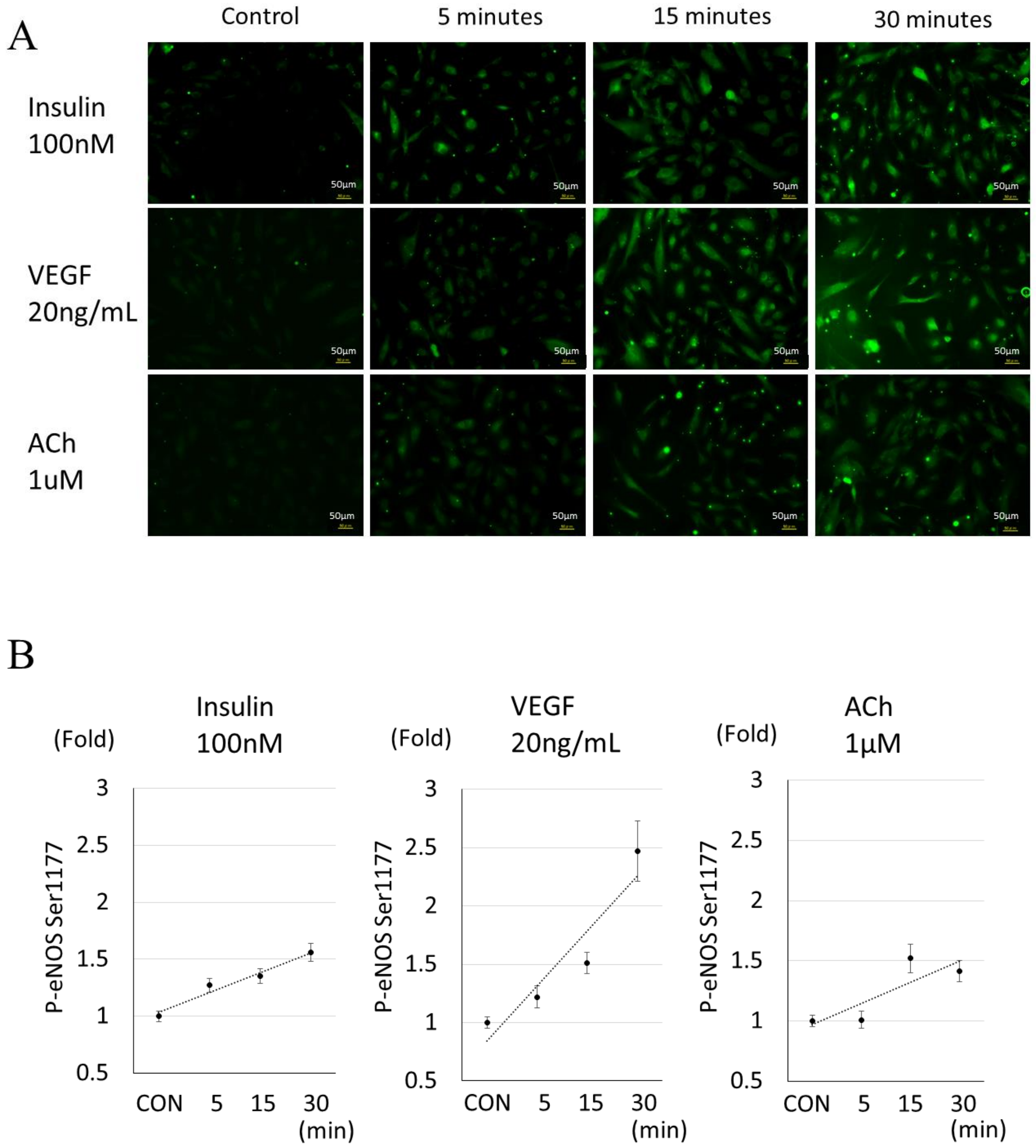

A: The immunofluorescent images with anti-p-eNOS antibody (1:200, GTX50212). Control slides had no stimulation. The times after each stimulation were indicated above the pictures.

B: The graphs of the intensities (ratio to average of control, mean ± standard error).

VEGF, vascular endothelial growth factor; ACh, acetylcholine.

Figure S4. The relationship between intensities of western blotting and immunofluorescence of HUVECs after insulin-stimulation.

A

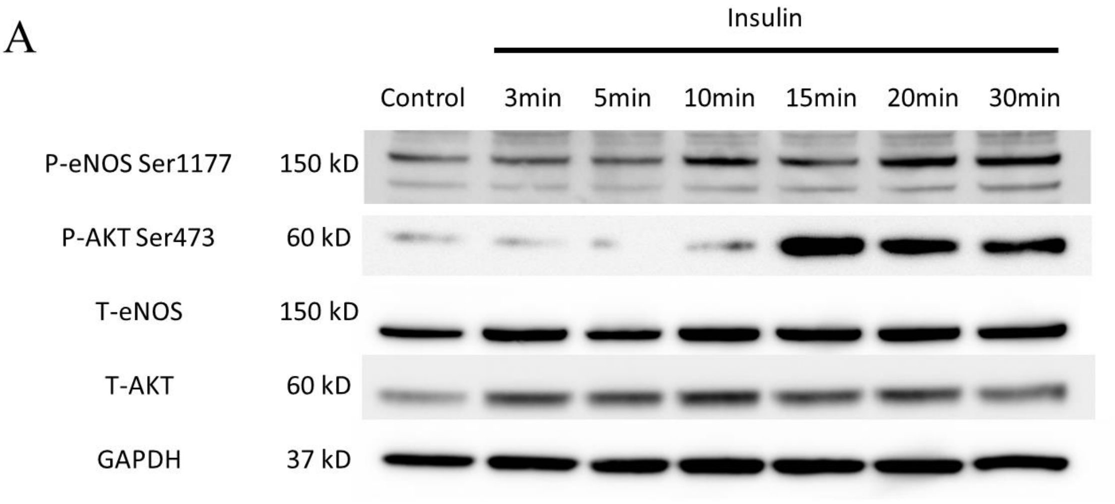

B

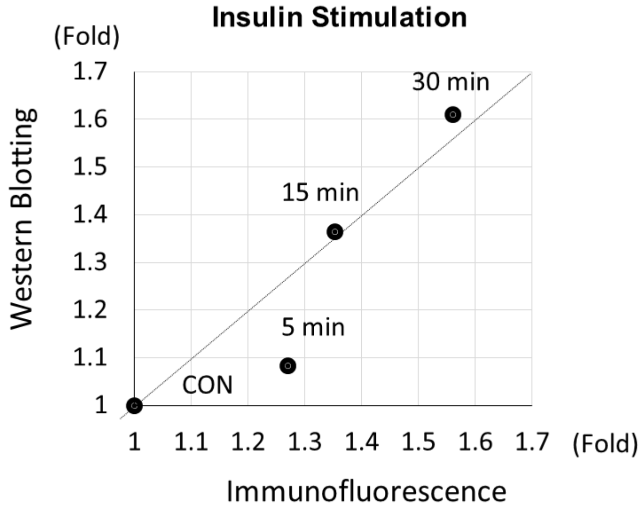

The immunoblotting image (A) shows gradual increase of p-eNOS Ser1177 after rise of p-Akt Ser473 by addition of insulin 100 nM. The plots (B) shows the

positive correlation of the results from western blotting and immunofluorescence.

Figure S5. Elimination of p-eNOS Ser1177 by eNOS knockdown.

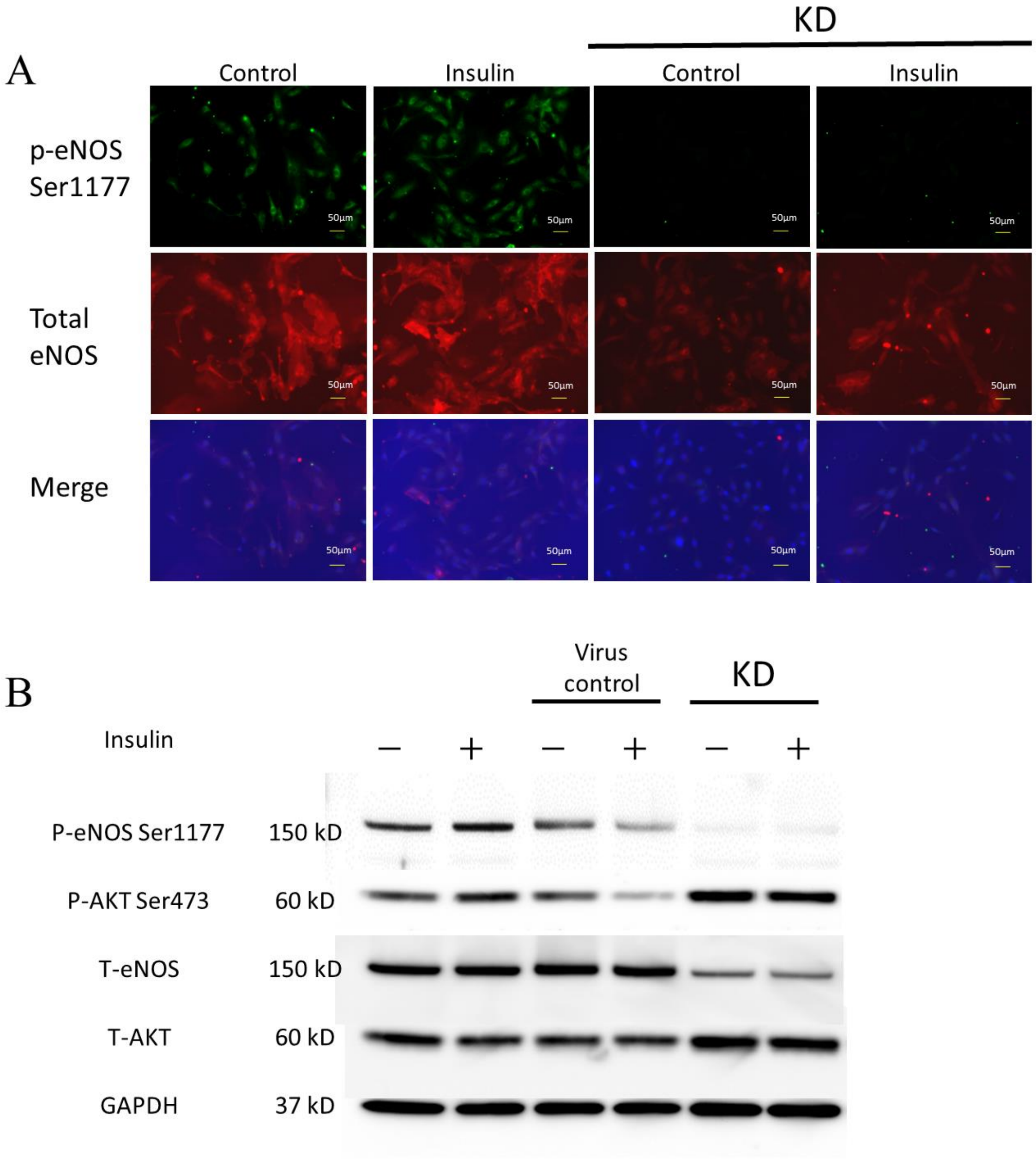

A: The immunofluorescent images with anti-p-eNOS antibody (GTX50212). B: The western blotting. Control slides had no stimulation. Insulin simulation was 100 nM 30 minutes. KD, eNOS knockdown.

Figure S6. Augmentation of p-eNOS Ser1177 by serum-stimulation.

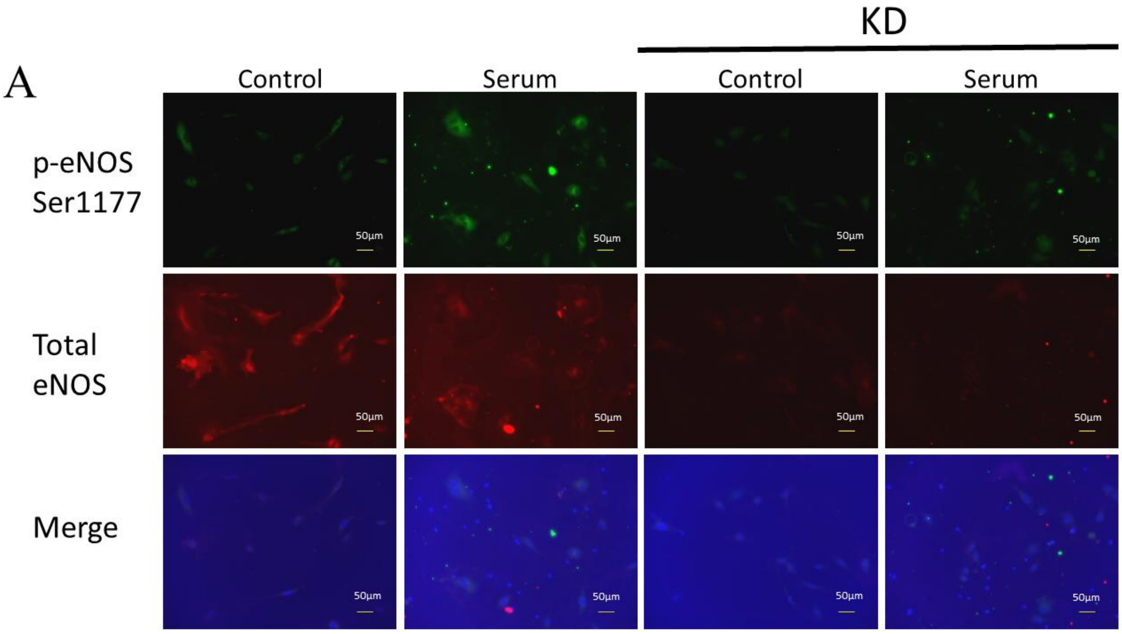

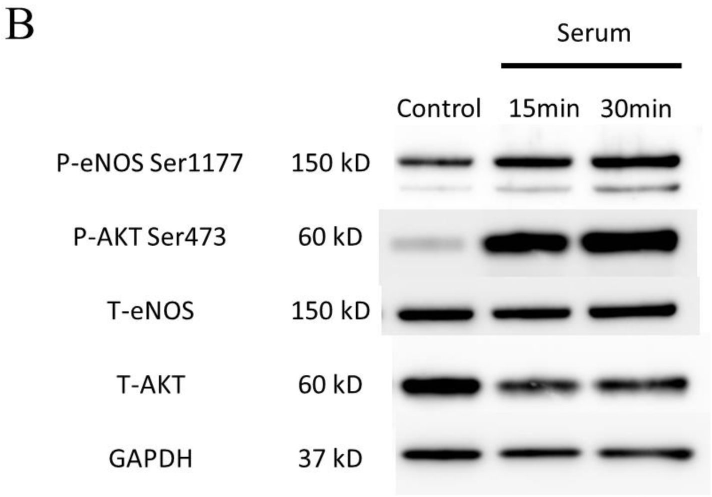

C

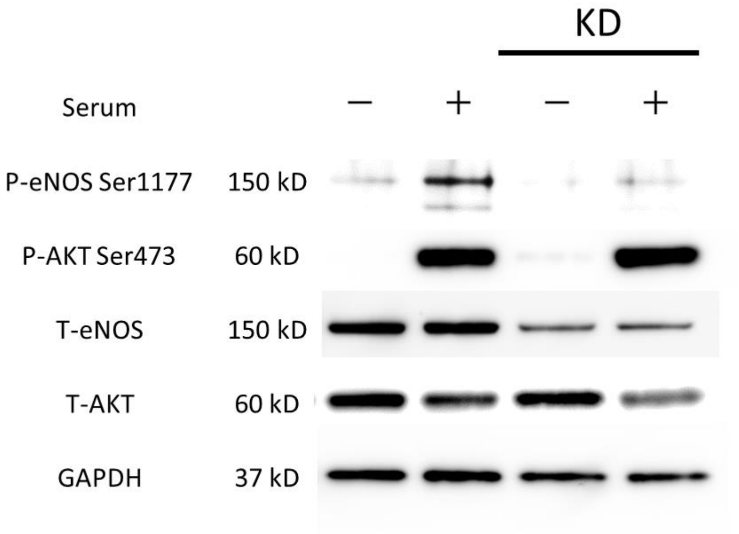

D

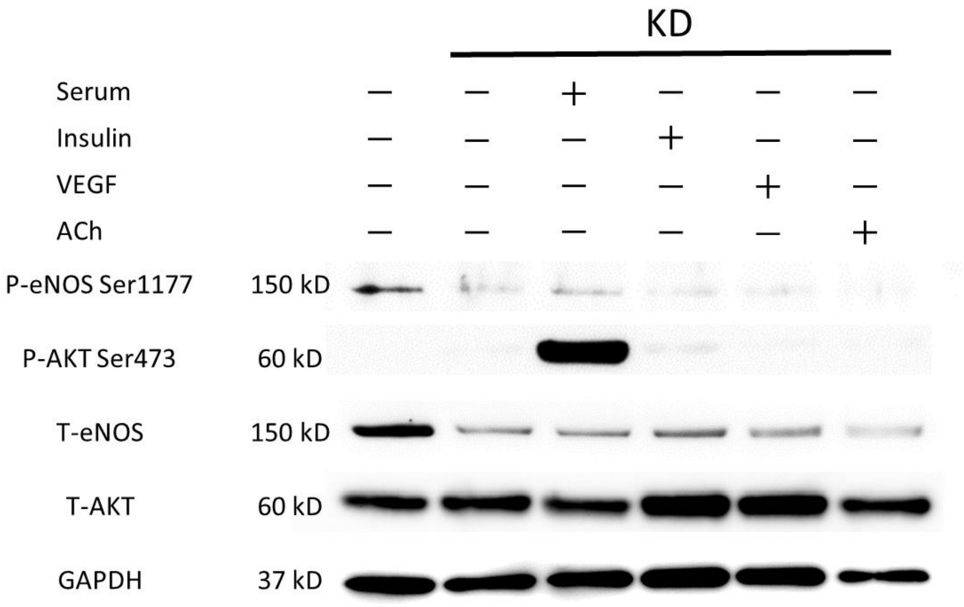

A: The immunofluorescent images with anti-p-eNOS antibody (GTX50212). B: The western blotting of time-course p-eNOS Ser1177 after serum-stimulation. C: The western blotting showing the effects of KD on HUVECs with serum-

stimulation. D: The western blotting showing the effects of KD on HUVECs with other stimulations.

Control slides had no stimulation. Serum-stimulation was incubating with human fresh serum for 30 minutes. Insulin-simulation was addition of insulin 100 nM for 30 minutes. VEGF-stimulation was 20 ng/mL for 30 minutes. ACh-stimulation was 1 μM for 15 minutes.

KD, eNOS knockdown; VEGF, vascular endothelial growth factor; ACh, acetylcholine.

Figure S7. Solution path.

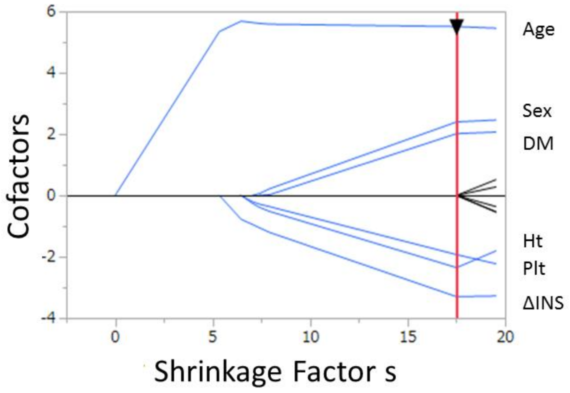

The original prediction formula was as follows; 7.347962 + (-0.015367)*ΔINS + 0.0831406*Age + 0.869302*Sex + 0.6601768* DM + (-0.086475)* Ht + (-0.043499)* Plt.

The categorical variables are Sex (man 1, woman 0) and DM (yes 1, no 0).

ΔINS, percent change in insulin-induced p-eNOS at Ser1177; DM, diabetes mellitus; Ht, hematocrit; Plt, platelets.

Supplemental References:

1. Tabit CE, Shenouda SM, Holbrook M, Fetterman JL, Kiani S, Frame AA, Kluge MA, Held A, Dohadwala MM, Gokce N, Farb MG, Rosenzweig J, Ruderman N, Vita JA, Hamburg NM. Protein kinase C-β contributes to impaired endothelial insulin signaling in humans with diabetes mellitus. *Circulation.* 2013;127:86-95.

2. Bretón-Romero R, Feng B, Holbrook M, Farb MG, Fetterman JL, Linder EA, Berk BD, Masaki N, Weisbrod RM, Inagaki E, Gokce N, Fuster JJ, Walsh K, Hamburg NM. Endothelial Dysfunction in Human Diabetes Is Mediated by Wnt5a-JNK Signaling. *Arterioscler Thromb Vasc Biol.* 2016;36:561-569.

3. Yamada T, Adachi T, Ido Y, Masaki N, Toya T, Uchimuro T, Nishigawa K, Suda H, Osako M, Yamazaki M, Takanashi S, Shimizu H. Preserved Vasoconstriction and Relaxation of Saphenous Vein Grafts Obtained by a No-TouchTechnique for Coronary Artery Bypass Grafting. *Circ J.* 2018;83:232-238.

4. Lan F, Cacicedo JM, Ruderman N, Ido Y. SIRT1 modulation of the acetylation status, cytosolic localization, and activity of LKB1. Possible role in AMP-activated protein kinase activation. *J Biol Chem.* 2008;283:27628-35.

5. Thors B, Halldórsson H, Jónsdóttir G, Thorgeirsson G. Mechanism of thrombin mediated eNOS phosphorylation in endothelial cells is dependent on ATPlevels after stimulation. Biochim Biophys Acta. 2008;1783:1893-902.

6. Motley ED, Eguchi K, Patterson MM, Palmer PD, Suzuki H, Eguchi S. Mechanism of endothelial nitric oxide synthase phosphorylation and activation by thrombin. *Hypertension.* 2007;49:577-83.

7. Touyz RM. Regulation of endothelial nitric oxide synthase by thrombin. *Hypertension*. 2007;49:429-31.
